# Systematic noise degrades gene co-expression signals but can be corrected

**DOI:** 10.1186/s12859-015-0745-3

**Published:** 2015-09-24

**Authors:** Saskia Freytag, Johann Gagnon-Bartsch, Terence P. Speed, Melanie Bahlo

**Affiliations:** 1 Bioinformatics Division, Walter + Eliza Hall Institute, 1G Royal Parade, Melbourne, 3050 Australia; 2 Department of Mathematics and Statistics, University of Melbourne, Melbourne, 3010 Australia; 3 Department of Statistics, University of California, 367 Evans Hall, Berkeley, 94720 USA; 4 Department of Medical Biology, University of Melbourne, Melbourne, 3010 Australia

**Keywords:** Gene co-expression, Data cleaning, Removal of unwanted variation, Human brain, Epilepsy

## Abstract

**Background:**

In the past decade, the identification of gene co-expression has become a routine part of the analysis of high-dimensional microarray data. Gene co-expression, which is mostly detected via the Pearson correlation coefficient, has played an important role in the discovery of molecular pathways and networks. Unfortunately, the presence of systematic noise in high-dimensional microarray datasets corrupts estimates of gene co-expression. Removing systematic noise from microarray data is therefore crucial. Many cleaning approaches for microarray data exist, however these methods are aimed towards improving differential expression analysis and their performances have been primarily tested for this application. To our knowledge, the performances of these approaches have never been systematically compared in the context of gene co-expression estimation.

**Results:**

Using simulations we demonstrate that standard cleaning procedures, such as background correction and quantile normalization, fail to adequately remove systematic noise that affects gene co-expression and at times further degrade true gene co-expression. Instead we show that a global version of removal of unwanted variation (RUV), a data-driven approach, removes systematic noise but also allows the estimation of the true underlying gene-gene correlations. We compare the performance of all noise removal methods when applied to five large published datasets on gene expression in the human brain. RUV retrieves the highest gene co-expression values for sets of genes known to interact, but also provides the greatest consistency across all five datasets. We apply the method to prioritize epileptic encephalopathy candidate genes.

**Conclusions:**

Our work raises serious concerns about the quality of many published gene co-expression analyses. RUV provides an efficient and flexible way to remove systematic noise from high-dimensional microarray datasets when the objective is gene co-expression analysis. The RUV method as applicable in the context of gene-gene correlation estimation is available as a BioconductoR-package: RUVcorr.

**Electronic supplementary material:**

The online version of this article (doi:10.1186/s12859-015-0745-3) contains supplementary material, which is available to authorized users.

## Background

With the advent of affordable high-throughput technologies, numerous gene expression studies involving large numbers of samples have been conducted. This development inspired ongoing research into analysis tools that allow the systematic interrogation of gene organization using expression data. Gene co-expression methods are one of the better-known examples of such tools. They have been routinely used in the construction of biological pathways (i.e. [1]), gene annotation [2, 3], and even for assessing preservation of biological systems across different species [4]. Gene co-expression methods equate dependence between expression levels of two genes with the presence of a potential functional interaction. While there are several statistically appropriate and arguably better ways to measure such dependence, the Pearson correlation coefficient (PCC) remains the most widely adopted.

A weakness of the PCC is its inaccuracy when pairs of observations are not independent. Even though this problem is well known in statistics [5–8], it is often overlooked in the context of high-dimensional gene expression studies where sampling and the data generation process can produce dependencies. In the most extreme case, unaccounted dependencies between samples lead to an almost random sign in the PCC [9]. Consequently, in the presence of sample dependencies the PCC displays a high false-positive rate as well as a high false-negative rate of identified gene-gene relationships. Unfortunately, for large gene expression studies dependencies between samples are the norm. These are introduced by systematic noise, whose sources are often unknown.

The sources of systematic noise in gene expression studies are plentiful, arising from biological and technical factors [10]. A much-discussed example is the batch effect. Interestingly, batches themselves do not constitute the actual cause, rather technical or biological differences between batches result in systematic noise. The sheer scale of large gene expression studies means the introduction of systematic noise is very hard to avoid. Systematic noise is typically introduced through batch effects or sources, such as the involvement of several research centers, different sample sources and/or platforms. Moreover, it is generally unlikely for large gene expression studies that all sources of systematic noise are identified and directly measured. Indeed, in our experience the accompanying sample documentation of large gene expression studies is often partially or completely missing making an a priori investigation of potential sources of noise even more difficult.

Both a direct and an indirect approach for dealing with systematic noise when measuring gene co-expression in high-dimensional gene expression studies exist. The direct approach involves accounting for sample dependencies during the estimation of gene co-expressions. Examples include the mixed model based correlation measures of Teng and Huang [9] as well as more recently an approach by Jauhiainen et al. [11]. In contrast, the indirect approach first attempts to remove the systematic noise and then applies the PCC (or other measures of co-expression). A direct approach is generally preferable, however to our knowledge no generally applicable approach exists yet. The approach by Teng and Huang relies on replicate samples, which might not always be available, while the approach by Jauhiainen et al. is currently only working for small numbers of genes. Finally, the indirect approach with its cleaning step and correlation estimation step is more natural in the world of gene expression studies, because it has the benefit of flexibility. For example, more complex analytical tools, such as gene network construction methods, can still be applied.

A large number of methods for the removal of systematic noise in gene expression data have been proposed and compared. However, to our knowledge, these have never been systematically studied when the goal is the estimation of gene co-expression. Indeed, many of the existing methods, such as surrogate variable analysis [12] and ComBat [13], are unsuitable in this context. These methods depend on knowing the factor of interest, as is the case for differential expression analysis. Other tools, like distance weighted discrimination [14], crucially use information on batches in order to clean the data. Since information on batches is not always available for large gene expression studies and may not reflect the main sources of noise, such tools are likewise inadequate. Therefore, until recently, researchers relied on procedures focusing on the removal of scaling effects, such as quantile normalization (QN) [15], or used raw data when estimating gene co-expression. Such standard cleaning approaches have in the past been criticized, as they tend to degrade the correlation structure of gene expression signals [16, 17].

The lack of suitable cleaning procedures that remove systematic noise without the need for a factor of interest and/or sample documentation, led Jacob et al. [18] to develop naive RUV-random, an effective data driven procedure (“RUV” stands for “remove unwanted variation”). Unlike procedures removing only scaling effects, naive RUV-random can deal with multivariate noise behavior, i.e. situations where genes are not equally affected by systematic noise. We believe that this makes naive RUV-random much better suited at removing unwanted dependencies between samples that are introduced by systematic noise and can lead to inaccurate PCC estimates. Like RUV-2 [10], naive RUV-random makes use of so-called negative control genes. Negative control genes are genes that are assumed to be corrupted by the systematic noise, but that also, crucially, do not exhibit any biological variation of interest. Any variation observed in the negative control genes can therefore be assumed to be noise, and the negative controls can thus be used to learn the structure of this noise. Unlike RUV-2, which is intended for use only in differential expression analyses, naive RUV-random is intended for more general use, including the estimation of gene co-expression, which has however not been investigated until now. Nevertheless, it should be noted that, relative to RUV-2, naive RUV-random is fairly sensitive to confounding between the biological factor of interest and the systematic noise.

In this paper, we demonstrate that naive RUV-random allows improved estimation of co-expression from gene expression data via the PCC. In particular, we expose some shortcomings of the frequently employed data cleaning strategy of combining background correction (BC) [19] and QN when the interest is gene co-expression. To this end, we firstly conducted a simulation study; the results of which can be replicated using our R-package RUVcorr. The package also implements the naive RUV-random method, modified for gene co-expression analyses, and incorporates useful visualization tools. The simulation study examines the behavior of the cleaning strategies under the null hypothesis of no gene co-expression and sets of genes with gene co-expression that vary in their dependence with systematic noise. Secondly, we apply our method and competing methods to five large, previously published data sets of gene expression in normal human brains of foetuses, children and adults [20–24]. Since these datasets differ in sample size, design, platform, etc. we believe that they provide a suitably thorough testing ground.

In the absence of a known truth for the real data, we largely assess the performance of different cleaning approaches by the consistency of their results across the five data sets. Here, results refer to candidate epileptic encephalopathy (EE) genes prioritized using a method based on the PCC similar to the one used in the publication by Oliver et al. [25]. EEs are a group of devastating epilepsies whose genetically heterogeneous basis has yet to be fully understood. With the help of naive RUV-random, we can identify the most promising genes in the candidate list that should be the focus of further EE studies. Importantly, some of the identified genes were recently independently validated as true EE genes.

The remainder of this article is organized as follows. The ‘Results’ section presents findings from the simulation study and the application to the five real datasets. In the ‘Discussion’ section we conclude by discussing the implications of our results and the necessary changes in the approach to analyzing gene expression data when aiming to comprehend gene organization. Finally, the ‘Methods’ section describes naive RUV-random in the context of estimating gene co-expression as well as providing some remarks on the practical application of the procedure. Note that for simplicity instead of naive RUV-random we simply use RUV-random for the remainder of this article. We also give descriptions of the real datasets that were used.

## Results

### Results of the simulation study

The simulations were conducted using the linear model of biological signal plus systematic noise plus random noise, which is proposed by the RUV framework. While real data may not reflect this model perfectly, we propose that these simulations will shed light on the properties of different cleaning approaches. In particular, we compared using raw simulated data, simulated data cleaned with background-correction (BC), simulated data cleaned with BC and quantile-normaliztion (QN) and simulated data cleaned with RUV-random. Each method was applied to 1000 simulations, each with 500 genes with known correlation structure. (For more information of the simulation design refer to Additional file 1). In order to evaluate whether correlation estimates are close to the truth, we use the percentage of estimates with the wrong sign (WS) and a norm similar to the squared Frobenius norm:

$$\text{FN}^{2}=\frac{2\sum_{i=1}^{n}\sum_{j=i+1}^{n} \left(\text{arctanh} (\hat{r}_{i,j})- \text{arctanh} (r_{i,j})\right)^{2}}{n(n-1)}, $$

where *n* is the number of genes that are considered. The term $\hat {r}_{i,j}$ refers to the estimated PCC for genes *i* and *j*, while *r*_*i*,*j*_ refers to the true correlation. Note that this norm can be interpreted as the squared average error of the Fisher z-transformed correlation estimates.

We first examined the behavior of the different approaches under the null hypothesis of no true correlation between genes (Table 1). While cleaning simulation data with BC and BC in combination with QN (BC+QN) offered little improvement over the raw data, correlation estimates were closer to the truth for simulation data treated with RUV-random. We next investigated non-null simulation scenarios where genes were moderately correlated with each other. We observed similar results as under the null hypothesis (Table 2). Nearly 50 % of the correlation estimates had the wrong sign for standard approaches. In contrast RUV-random produced correlations that had the wrong sign for less than 1 % of correlation estimates. We also tried combining RUV-random with the two standard cleaning approaches with little success. Overall RUV-random applied to the raw simulation data, without BC or QN, clearly yielded the best results (see Additional file 1). However, it should be noted that we did not simulate measured background noise. It is conceivable that in some cases the removal of such measured background noise could also be beneficial prior to the application of RUV-random.

**Table 1:** Performance of different cleaning approaches when there is no genuine correlation between genes (based on 1000 simulation runs)

Method	Raw	BC	BC+QN	RUV-random
FN^2^	0.126	0.124	0.125	0.001

The performance was measured using a measure similar to the squared Frobenius norm (FN^2^) measure (explained in the text), which should be close to 0. All parameter choices and details of the simulation can be found in Additional file 1. All standard deviations were <0.001

**Table 2:** Performance of different cleaning approaches when there is moderate genuine correlation between genes (based on 1000 simulation runs)

Method	Raw	BC	BC+QN	RUV-random
% WS	48.2	48.1	48.0	0.7
FN^2^	0.423	0.420	0.414	0.005

The performance was measured using the proportion of estimates with the wrong sign (WS). While FN^2^ refers to a squared Frobenius norm (FN^2^) measure (explained in the text), which should be close to 0. All parameter choices and details of the simulation can be found in Additional file 1. All standard deviations for FN^2^ were <0.001

As BC and BC+QN exhibited inferior performance than RUV-random for our simulations, we concentrated on the properties of RUV-random. In particular, we explored the performance of RUV-random when the absolute values of correlations between genes were varied and when correlations between signal and noise are introduced. Table 3 shows that the absolute value of the simulated gene-gene correlations did not influence the performance of naive RUV-random. However the ability to correctly estimate gene-gene correlations deteriorated with increasing correlation between signal, *X*, and systematic noise, *W* (see Table 4), which is expected. Note that further simulation scenarios concerning the choice of negative control genes can be found in Additional file 1.

**Table 3:** Performance of RUV-random for different average absolute value of gene-gene correlations (based on 1000 simulation runs)

Average gene-gene correlation	% WS	FN^2^
0.2586	2.0	0.006
0.2905	1.4	0.005
0.3384	0.9	0.004
0.4225	0.5	0.008

The performance was measured using the percentage of estimates with the wrong sign (WS). While FN^2^ refers to a squared Frobenius norm (FN^2^) measure (explained in the text), which should be close to 0. All parameter choices and details of the simulation can be found in Additional file 1. All standard deviations for FN^2^ were <0.001

**Table 4:** Performance of RUV-random when biological signal and systematic noise are differently correlated (based on 1000 simulation runs)

Average Cor(*W*,*X*)	% WS	FN^2^
0.1127	8.3	0.138
0.1634	14.5	0.211
0.2015	21.6	0.281
0.2341	27.7	0.341

The performance was measured using the percentage of estimates with the wrong sign (WS). While FN^2^ refers to a squared Frobenius norm (FN^2^) measure (explained in the text), which should be close to 0. All parameter choices and details of the simulation can be found in Additional file 1. All standard deviations for FN^2^ were <0.001

### Results of the real data application

We generated the following four versions for each of the five real datasets:

1. raw, non-normalized data (Raw)
2. background-corrected data (BC) [19]
3. data treated with a combination of background-corrected and quantile-normalized (BC+QN) [15]; this method is favored during the pre-processing of many gene expression studies [26] (Note that for the Colantuoni et al. study we applied background-correction in combination with loess-normalization (BC+LN) [27] instead.)
4. data cleaned using the outlined RUV-random approach (RUV-random) (for details see Additional file 1) (Note that for the Hernandez et al. study we mostly used background-correction in combination with RUV-random (BC+RUV-random) for reasons explained below.)

Note that for both the Hawrylycz et al., Miller et al. and Hernandez et al. studies we also included their normalized data in our comparison, as they use a data normalization approach beyond BC+QN [24, 28].

#### Comparison using correlation densities

Since for real datasets the truth is unknown, it is difficult to evaluate the performance of different microarray data cleaning procedures. However, genes with known correlation structure provide one way to judge performances. Using histograms or smoothed density plots of the correlation of such genes we can check whether differently cleaned versions of the data result in the expected correlation structures. Firstly, we use a set of 1000 randomly sampled genes that we expected to mostly have correlations about 0. In fact, given a large enough set of random genes the density of their correlations is expected to be a roughly normal distribution centered around 0, i.e. mode 0. Ploner et al. [17] made a similar assumption in their work on the effect on low-level standardization of correlation estimates; noting that any random sample of pairs of genes ‘[...] will contain only a small percentage of unequivocal biological relationships’. Secondly, we used a set of genes involved in the polycomb repressor complex 2 (PRC2). These genes, *EED*, *EZH2*, *RBBP4*, *RBBP7* and *SUZ12*, are known to be tightly co-regulated, as they are required for long term epigenetic silencing of chromatin in all multicellular organisms. Thus, we expect strong positive correlations between these genes.

**Fig. 1:**
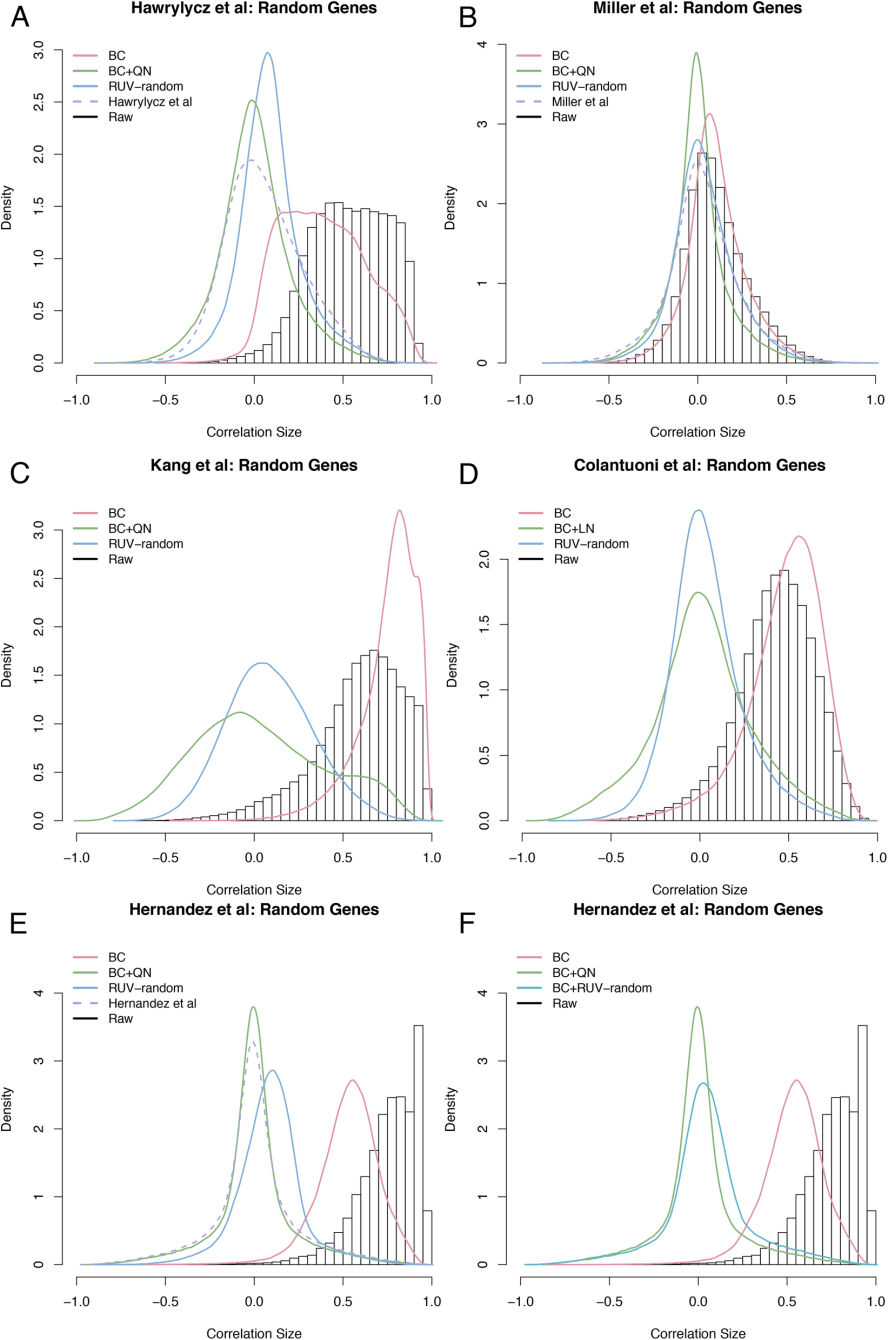
Density curves of the correlation between random genes for differently cleaned versions of all datasets. **a** Correlation density curves as estimated from the raw, BC, BC+QN and RUV-random versions of the Hawrylycz et al. data. The dashed line (purple) represents the density of the correlations between random genes estimated from the version of the data normalized by the authors. **b** Correlation density curves estimated from the raw, BC, BC+QN and RUV-random versions of the Miller et al. data. The dashed line (purple) represents the density of the correlations between random genes estimated from the version of the data normalized by the authors. **c** Correlation density curves estimated from the raw, BC, BC+QN and RUV-random versions versions of the Kang et al. data. **d** Correlation density curves estimated from the raw, BC, BC+LN and RUV-randomversions of the Colantuoni et al. data. **e** Correlation density curves estimated from the raw, BC, BC+QN and RUV-random versions of the Hernandez et al. data. The dashed line (purple) represents the density of the correlations between random genes estimated from the version of the data normalized by the authors. **f** Correlation density curves estimated from the raw, BC, BC+QN and BC+RUV-random versions of the Hernandez et al. data. Note that here we display BC+RUV-random instead of RUV-random. The histogram in the background of all panels represents the density generated when using the raw version of each dataset

When using raw data correlations between pairs of random genes generally resulted in strongly positive correlations (compare Fig. 1) implying that systematic noise inflates gene co-expression estimates. The only exception was the Miller et al. dataset where the density of the correlation values of pairs of random genes was only slightly shifted to the right of zero. The application of BC mostly resulted in positive correlations, even though for most datasets these were decreased compared to the raw data. The exception was the Kang et al. dataset where we observed correlation values to increase further. The BC+QN (or BC+LN) versions of all datasets, except the Kang et al. study, resulted in correlation density curves tightly centered around 0.

RUV-random treated data resulted in correlation density curves that overall fitted our expectations best. In particular, the density curves were often tighter and more accurately centered around 0. The exception is the correlation density curve generated with the RUV-random version of the Hernandez et al. study. Because the curve was shifted to the right, we combined BC and RUV-random (BC+RUV-random) to clean this dataset. Interestingly, the density curves produced by both BC+RUV-random and BC+QN had roughly the same shape as the authors’ own normalization. The same also held in the case of the Miller et al. study, where RUV-random produced a very similar density curve to the authors’ own normalization.

**Fig. 2:**
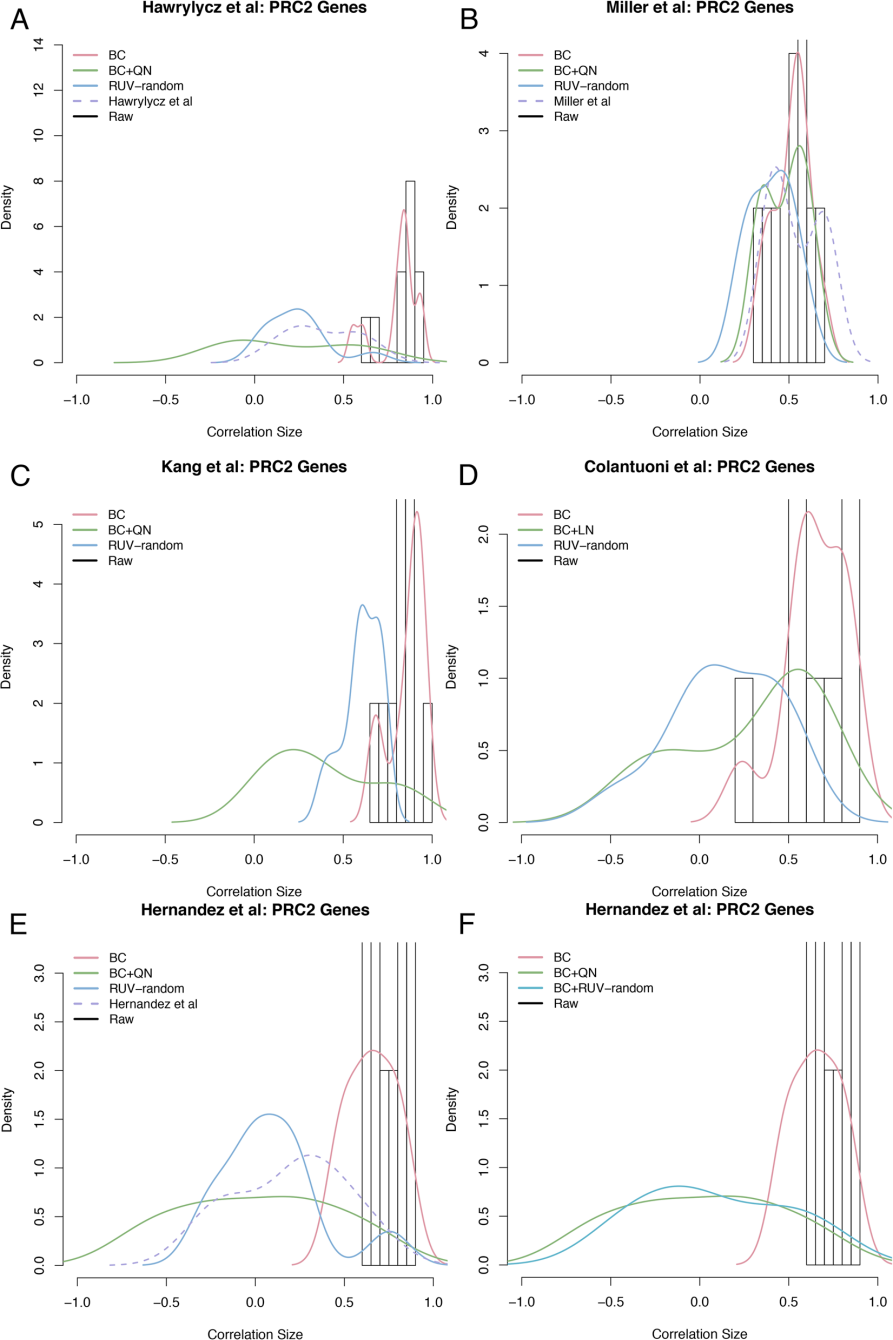
Density curves of the correlation between PRC2 genes for differently cleaned versions of all datasets. **a** Correlation density curves as estimated from the raw, BC, BC+QN and RUV-random versions of the Hawrylycz et al. data. The dashed line (purple) represents the density of the correlations between random genes estimated from the version of the data normalized by the authors. **b** Correlation density curves estimated from the raw, BC, BC+QN and RUV-random versions of the Miller et al. data. The dashed line (purple) represents the density of the correlations between random genes estimated from the version of the data normalized by the authors. **c** Correlation density curves estimated from the raw, BC, BC+QN and RUV-random versions versions of the Kang et al. data. **d** Correlation density curves estimated from the raw, BC, BC+LN and RUV-randomversions of the Colantuoni et al. data. **e** Correlation density curves estimated from the raw, BC, BC+QN and RUV-random versions of the Hernandez et al. data. The dashed line (purple) represents the density of the correlations between random genes estimated from the version of the data normalized by the authors. **f** Correlation density curves estimated from the raw, BC, BC+QN and BC+RUV-random versions of the Hernandez et al. data. Note that here we display BC+RUV-random instead of RUV-random. The histogram in the background of all panels represents the density generated when using the raw version of each dataset

Studying correlation density of PRC2 gene pairs supported that RUV-random is generally able to retrieve genuine gene-gene correlations (compare Fig. 2). For most datasets, we found density curves as estimated from the RUV-random version of the datasets displaying density curves that were situated in the positive domain in line with our expectations. The exceptions are the Colantuoni et al. and the Hernandez et al. datasets, which demonstrated wide distributions spanning the entire domain but with heavy right tails. In comparison the density curves as estimated from BC+QN versions of the datasets nearly always gave wider distributions. Even though the BC and raw versions of all datasets showed the strongest positive correlations, in most cases these density curves are very similar to the density curves estimated from correlations between random pairs of genes.

#### Comparison using empirical cumulative distribution functions

A second option to evaluate the performance of different cleaning strategies is to compare the empirical cumulative distribution functions (ECDF) curves of the absolute value of the gene-gene correlations of positive and negative control. If the cleaning procedure applied to the data works well then we expect an easily visible difference between the ECDF curves of the set of random genes and a set of known EE genes. Like the PRC2 genes, we expect the EE genes to be co-expressed as they are potentially part of the same disease pathway.

Except for the Hawrylycz et al. dataset of developing brain, RUV-random corrected data showed the biggest difference between the ECDF curves of EE genes and random genes amongst all investigated cleaning procedures (see Figures in Additional files 2–6). However, the disparity between the various cleaning procedures strongly depended on the technology and the study design. The application of RUV-random results in the biggest improvements for the Kang et al. study, which was generated using Affymetrix technology. There is also a clear improvement in the signal from the RUV-random corrected data compared to the other cleaning procedures for the custom two-color microarray used in the Colantuoni et al. study. The Hawrylycz et al. dataset produced with an Agilent custom array and the Hernandez et al. dataset produced with a commercial Illumina array also showed bigger separation between the ECDF curves of the random set of gene and the EE genes for the RUV-random treated or BC combined with RUV-random treated data. However, RUV-random did not show any improvement over BC+QN for the Miller et al. study, which was generated using a commercial Agilent array.

#### Comparison using *p*-value distributions

As a third comparison tool histograms of *p*-values obtained from testing the null hypotheses that the true correlations between random pairs of genes are zero. While we expect some of the correlations to be truly non-zero, and therefore their *p*-values to be significant, there should be many gene pairs that have 0 correlation. Ideally, their *p*-values should be uniformly distributed.

**Fig. 3:**
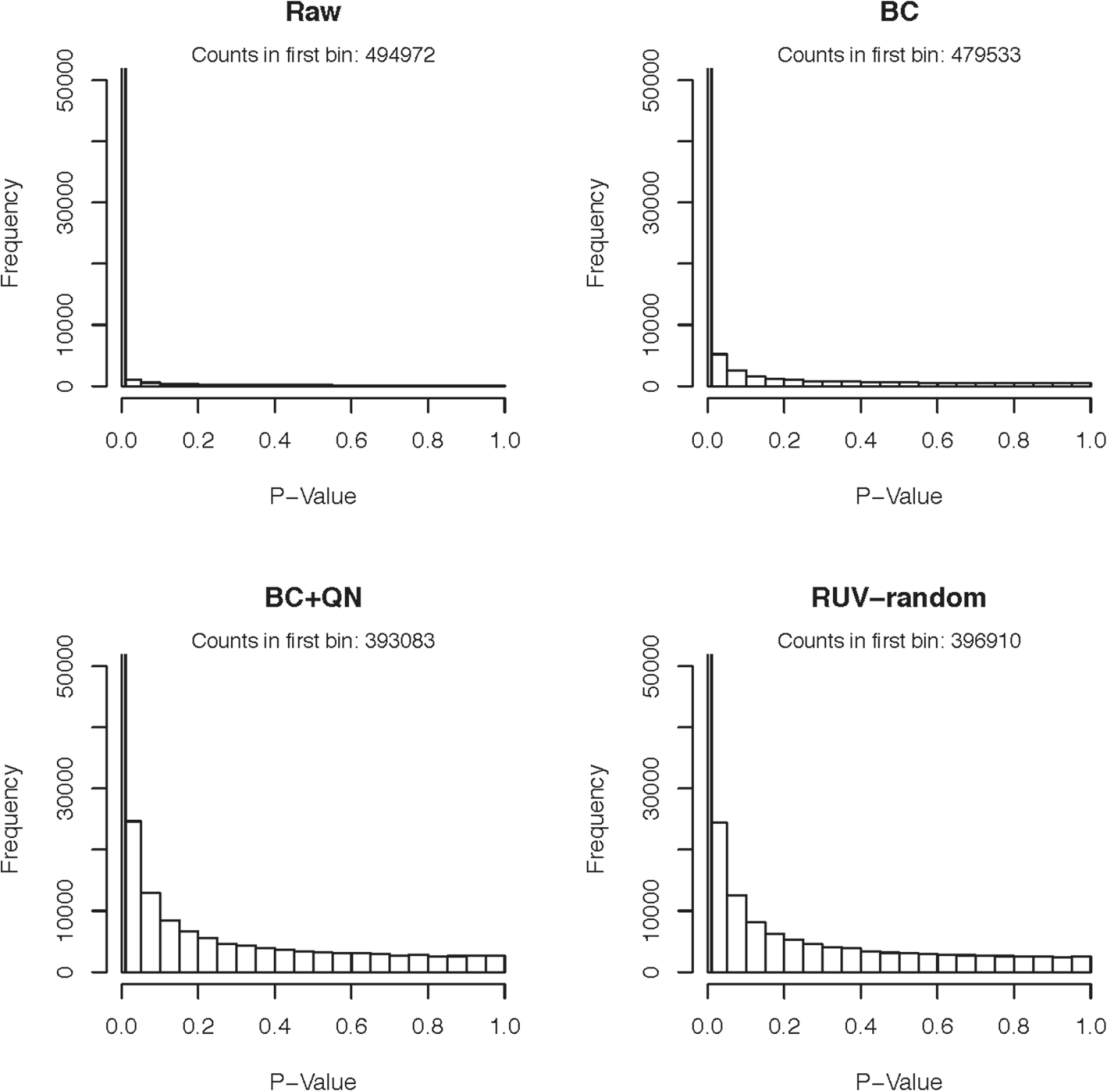
*P*-value histogram based on a t-test of the null hypotheses that the correlations between random pairs of genes in the Hawrylycz et al. dataset are zero. The count of the first bin [0−0.01) can be found in the caption of each plot

**Fig. 4:**
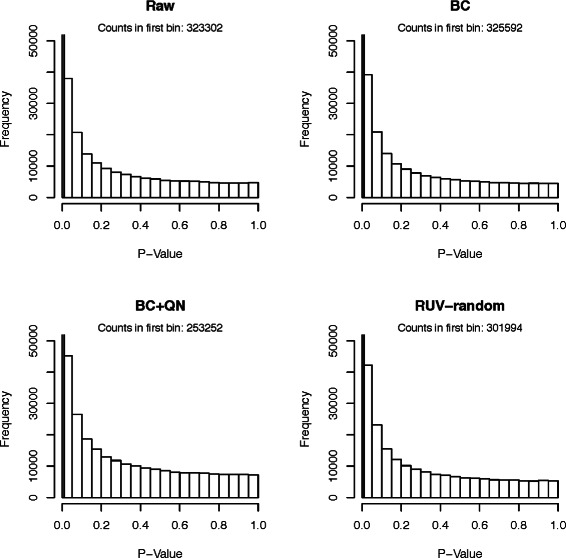
*P*-value histogram based on a t-test of the null hypotheses that the correlations between random pairs of genes in the Miller et al. dataset are zero. The count in the first bin [0−0.01) can be found in the caption of each plot

**Fig. 5:**
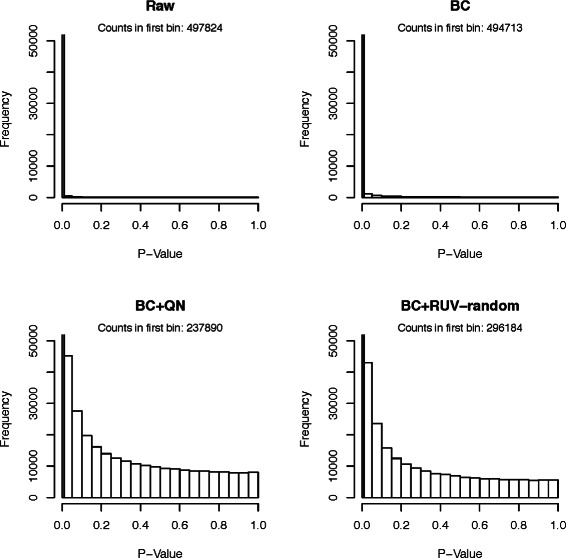
*P*-value histogram based on a t-test of the null hypotheses that the correlations between random pairs of genes in the Kang et al. dataset are zero. The count in the first bin [0−0.01) can be found in the caption of each plot

**Fig. 6:**
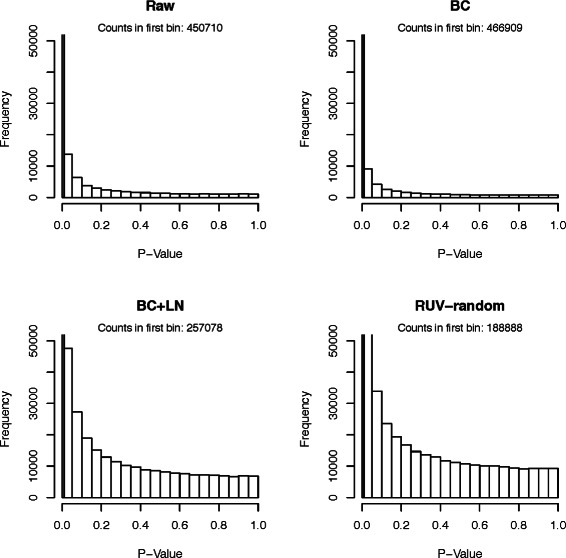
*P*-value histogram based on a t-test of the null hypotheses that the correlations between random pairs of genes in the Colantuoni et al. dataset are zero. The count in the first bin [0−0.01) can be found in the caption of each plot

**Fig. 7:**
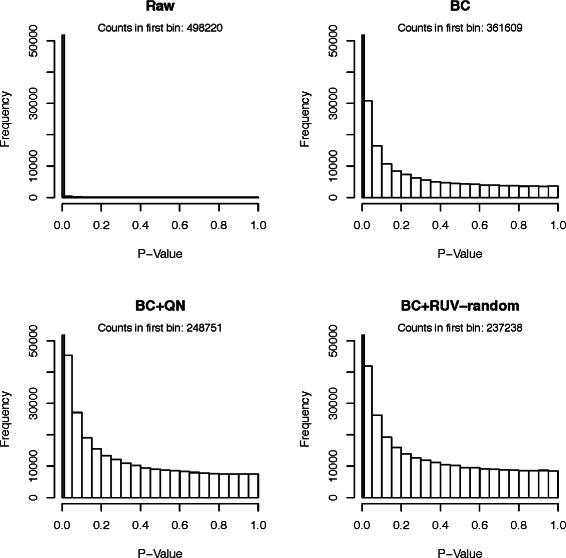
*P*-value histogram based on a t-test of the null hypotheses that the correlations between random pairs of genes in the Hernandez et al. dataset are zero. The count in the first bin [0−0.01) can be found in the caption of each plot

The *p*-value histograms of the raw version of all datasets showed extremely non-uniform distributions (compare Figs. 3, 4, 5, 6 and 7), for the Kang et al. the Colantuoni et al. and the Hernandez et al. studies the *p*-value histograms of the respective BC versions of the data did not demonstrate any improvement. The application of RUV-random resulted in *p*-value distributions that were closest to the uniform distribution for all studies. However, for the Hawrylycz et al. the Miller et al. and the Hernandez et al. datasets BC+QN and RUV-random produced very similar distributions.

#### Comparison using ranks of correlations

The fourth method for assessing performance focuses on individual correlations between genes known to be co-regulated. We used this approach because only a small proportion of genes will genuinely interact with each other, thus aggregate assessments can mask performance gains for this subset. To this end, we rank the correlations between all pairs of 1000 randomly selected genes and the EE genes (including the correlations between random genes and EE genes) according to their absolute value (highest to lowest). It is now possible to compare different cleaning approaches against a reference, such as the ranks calculated from the raw data. In particular, for every pair of EE genes the difference between its rank as calculated from the raw data and its rank calculated from a cleaned version of the data can be determined. If the cleaning procedure works, we expect a proportion of the ranks of the EE genes to be positive, indicating that their rank is lower when using the cleaned data than when using the raw data. We do not necessarily expect the ranks of all EE genes to decrease, as we only assume that the EE genes form a network and thus not all genes need to interact with each other.

**Fig. 8:**
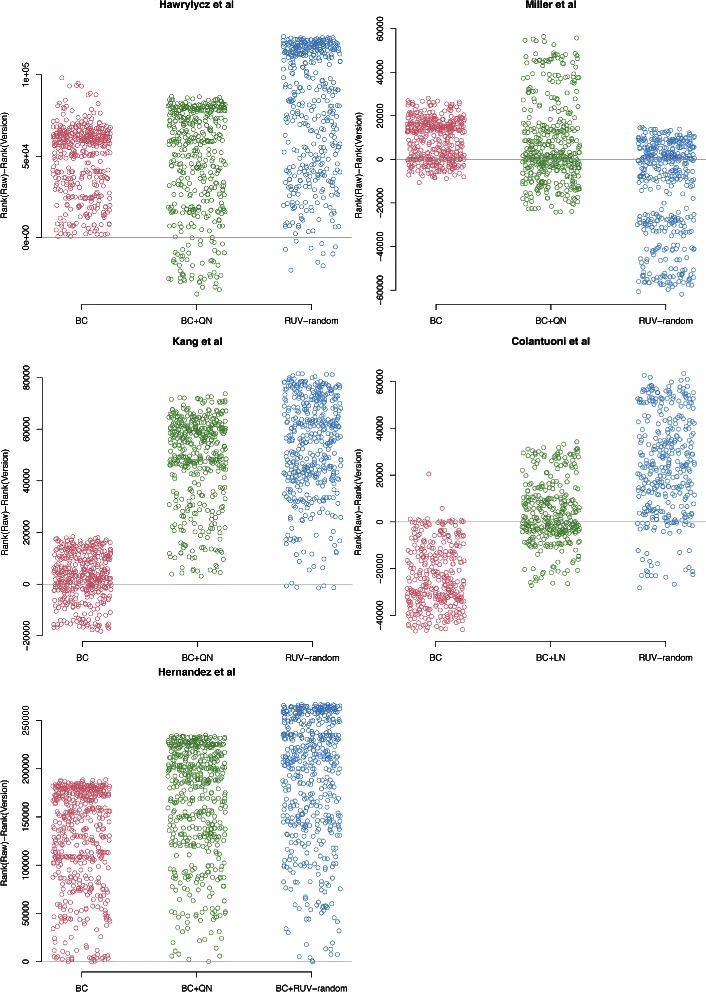
Scatterplots of the difference between ranks of EE gene pairs as obtained from the raw data and a different version of the data. The ranks were determined using the absolute value of the correlations of gene pairs obtained from 1000 randomly selected genes and EE genes. Each panel shows a different dataset

The difference between the ranks of EE gene pairs as determined from the raw data and the cleaned versions of the data supported our previous results (see Fig. 8). For all datasets except the Miller et al. dataset, ranks as determined by the RUV-random version of the data experienced the strongest decrease in ranks compared to ranks determined from the raw data. While not all pairs of EE genes displayed a stronger correlation compared to correlations between random pairs of genes after cleaning with RUV-random a significant proportion did. This indicates that the network of EE genes would have been more easily discovered when using the RUV-random version of the data.

#### Using prioritization to identify promising EE candidate genes

The final comparison tool we applied was *in silico* gene prioritisation, which allowed us to assess which cleaning method leads to greater consistency between studies. Gene prioritisation methods [29, 30] identify the most promising genes from a list of candidates lacking adequate support to corroborate their involvement in a specific disease pathogenesis. Establishing which genes are promising is important in order to effectively allocate resources for follow-up efforts, as it is often impossible to perform additional studies such as functional analysis or sequencing of many candidate genes in large cohorts for all candidates. The underlying concept of gene prioritization is referred to as “guilt-by-association”. Briefly, this concept supposes that all genes that are genuinely involved in the disease mechanism are part of the same or related biological networks. It should therefore be possible to identify disease genes in healthy tissue via gene co-expression, assuming that the co-expression occurs at the transcriptional level, which may not always be the case. To this end, many gene prioritization approaches utilize large, publicly available gene expression datasets, most often derived from transcriptomic analysis of lymphocyte derived RNA, the most easily obtained source of human RNA.

Here, we use gene prioritization of EE candidate genes in order to further illustrate the strength of the RUV-random cleaning procedure. At the time of the investigation (September 2014), there were 33 confirmed EE genes and 223 candidates, derived from two major sequencing studies. For more information on the choice of known EE and candidate EE genes see Additional file 1. We prioritized these candidates by applying a gene prioritization approach, which is largely the same as the one applied by Oliver et al. (for more details on the gene prioritization approach refer to Additional file 1) but using differently processed gene expression data. We compare the results of the prioritization results on the various versions of the datasets by assessing their consistency across different studies.

We demonstrated that the application of RUV-random (in case of the Hernandez et al. dataset BC+RUV-random) leads to greater consistency between studies. With RUV-random 7 of 160 shared gene candidates were prioritized in all five datasets. The next best performing method, BC+QN, only prioritized 3 genes in common between all five datasets (using the raw data we were also able to prioritize 3 candidates in all datasets). Note that for the BC version of the Kang et al. dataset prioritization was impossible, as we could not find a threshold to define significant correlations. When we excluded the Kang et al. dataset in order to also allow comparison with BC, RUV-random prioritized 11 candidates, while the next best method BC+QN only prioritized 9 genes in all four datasets. Furthermore, RUV-random treated data leads to the biggest number of prioritized candidates in 3 of 5 datasets (see Table 5). In one of the two other datasets, RUV-random only priortizes one gene less than the next best method. More details on prioritization results can be found in Additional file 1.

**Table 5:** Number of EE candidate genes prioritized in each version of each dataset. The prioritization method is discussed in detail in Additional file 1

Study	Raw	BC	BC+QN	RUV-random
Hawrylycz et al.	84	80	75	83
Miller et al.	80	82	84	86
Kang et al.	48	-^a^	33	62
Colantuoni et al.	33	42	43	44
Hernandez et al.	137	58	65	67^b^

^a^Prioritization for the background-corrected Kang et al. dataset was not possible as no cutoff value for significant correlations could be defined

^b^As discussed earlier, we use the version of BC+RUV-random for this dataset

## Discussion

The presence of systematic noise in large microarray gene expression datasets makes reliable inference of gene co-expression challenging. Frequently applied cleaning procedures, such as BC and QN, remove systematic noise in such a way that genuine gene-gene interactions may not be able to be retrieved from the data. Our simulations indicated that nearly half of the gene-gene correlation estimates, as computed via the PCC, had the wrong sign in the presence of moderate systematic noise. These results are supported by similar earlier findings for QN by Qiu et al. [16] and BC by Ploner et al. [17]. In order to overcome shortcomings of standard cleaning procedures researchers (for examples see Kang et al. [20] and Hawrylycz et al. [22]) often concentrate on a subset of the genes deemed to be more reliable. This strategy does not necessarily result in power reduction for global co-expression analysis. However, it can be detrimental for approaches like *in silico* prioritization, because genes pertaining to the research question might be excluded.

Here we described the usage of RUV-random in the context of estimating gene-gene correlations. RUV-random in the context of estimating gene-gene correlations is made available to others through the accompanying BioconductoR-package RUVcorr. Our simulation study demonstrated good performance in various scenarios, including scenarios where large proportions of negative control genes are wrongly specified. The results generated from the five datasets cleaned using RUV-random supported the simulation results. Applications of RUV-random to PRC2 genes known to have strong gene-gene correlations showed improved performance in comparison with standard normalization procedures. Comparison with the original approaches used in the studies, were not always possible as the normalization procedures applied were not suitable to correlation analysis or the respective data was not available.

The impact of RUV-random depends on the technology that produced the data as well as the amount of systematic noise. Treatment with RUV-random improved the Kang et al. dataset generated using the Affymetrix technology the most, out of all five datasets. In contrast, there were no benefits, but also no losses, when applying RUV-random to the Miller et al. study. This may be explained by the exceptionally high quality of the raw data. Creating such high quality data was possible because the researchers could draw on experience with protocols and technology gained during the Hawrylycz et al. study. In particular, they were able to reduce non-biological biases by using the same sample capturing method throughout the study (personal correspondence with the authors). Furthermore, randomization of samples across batches reduced sources of technical noise. The impact of RUV-random was also negligible for the Hernandez et al. dataset, which was generated using a commercial Illumina array. In combination with BC RUV-random did, however, produce convincing results for this dataset.

While RUV-random was always an improvement compared to BC, it did not necessarily give better results than BC+QN for all examined datasets. However, it did give the most consistent results across various different microarray technologies. This makes this cleaning procedure especially suited towards cross-study comparisons. This was also highlighted by the reproducibility of the RUV-random prioritization results of a set of candidate EE genes across all five datasets. No other method achieved such high reproducibility. Moreover, most of the prioritized EE candidate genes have since been independently replicated (see Additional file 1 for a full list). This includes the gene *DNM1*; a gene that was recently observed in five individuals with epileptic encephalopathy to harbor de novo mutations [31].

RUV-random only works well when it is applied with care to datasets with a minimum sample size of 100. Sample sizes smaller than this are not suitable for co-expression analysis in most cases. Investigators need to thoroughly examine their parameter choices using appropriate control genes. On the one hand, this complexity might discourage potential users. On the other hand, these parameters permit enormous flexibility as they let the investigator determine how aggressively the data is cleaned. This will depend on the research question. Furthermore, investigators should be aware that RUV-random breaks down when the genuine biological signal and the systematic noise are too correlated. However, at present no method exists that can deal with this complex situation. Since it is difficult or even impossible to know whether this is indeed the case for a dataset in question, this presents an important area for further research. Another important research area is the application of RUV-random to other types of data. In particular, it would be interesting to apply RUV-random to RNA-seq data. Unlike microarrays, RNA-seq data can reveal sequence variations and detect novel transcripts. In addition it is more comprehensive (subject to depth of sequencing), theoretically able to analyse all expressed genes and other RNA producing genomic regions. In principle, RNA-seq data, summarized to reads per kilobase per million should also be suitable for RUV-random correction, however there are still relatively few large RNA-seq datasets suitable for gene co-expression analysis.

Finally, the present paper raises concerns about the many already published papers and methods aiming to infer gene co-expression from microarray data. Unless the data is exceptionally free of systematic noise the results of such inferences have little in common with the underlying biological mechanisms. It is likely that this problem also affects newer technologies, like RNA-seq. Therefore we believe that the microarray community needs to change their approach to data cleaning in general. Instead of creating one “cleaned” version of the dataset, datasets should be cleaned with a particular objective in mind.

## Methods

### RUV focused on correlation estimation

The RUV procedure is based on the assumption that the data can be represented as the following multivariate linear model:

$$Y=X\beta+W\alpha+\epsilon $$

with $Y \in \mathbb {R}^{m \times n}$, $X \in \mathbb {R}^{m \times p}$, $\beta \in \mathbb {R}^{p \times n}$, $W \in \mathbb {R}^{m \times k}$, $\alpha \in \mathbb {R}^{k \times n}$ and $\epsilon \in \mathbb {R}^{m \times n}$. In other words, the gene expression on a log-2 scale of *n* genes for m samples, denoted by the matrix *Y*, can be expressed as a linear combination of the genuine signal, *X* *β*, some systematic noise, *W* *α*, and some random noise, *ε* (assumed to be $\epsilon _{j} \sim \mathit {N}(0, \sigma _{\epsilon }^{2}I_{m})$, for *j*=1,…,*n*). This model has been found to be effective in many studies, see [10]. The parameter *X* is also referred to as the factor of interest, where *p* denotes its dimensionality, which is unknown. The matrix *W* contains the *k* unobserved covariates that introduce systematic noise (note that *k* is also unobserved). The matrices *β* and *α* are unobserved coefficients that determine the influence of their relevant components on a particular gene.

In 2012, Gagnon-Bartsch and Speed [10] provided estimators for *W* and *α* in the case of known *X*. They exploit the fact that some genes are known to be unrelated to the factor of interest, so called negative controls:

$$Y_{c}=W\alpha_{c}+\epsilon_{c} $$

where the index *c* indicates the columns of the negative control genes. The matrix *W* is estimated by performing a factor analysis on *Y*_*c*_. The coefficients *β* and *α* are then estimated by regressing *Y* on *X* and $\hat {W}$. Jacob et al. [18] considered the case in which *X* is unobserved. In this case, *W* is still estimated by performing a factor analysis on *Y*_*c*_. However, since *X* is unobserved, it is no longer possible to estimate *β* and *α* by regressing *Y* on *X* and $\hat {W}$. Instead, to estimate *α*, *Y* is regressed on $\hat {W}$ alone. Then $\hat {W}\hat {\alpha }$ is subtracted from *Y* to produce cleaned dataset. Note that, because *Y* is regressed on $\hat {W}$ alone, the resulting estimate of *α* will be biased if *X* and *W* are correlated. If indeed *X* and *W* are correlated, the net effect of this bias is that some of the biological variation of interest will be removed along with the noise. This is a serious concern, particularly because the correlation of *X* and *W* is generally unknown in practice. To partially mitigate this problem, RUV-random uses ridge regression when estimating *α*. While this does not eliminate the potential for bias, it does allow the user to control how aggressively the noise — and possibly the signal — are to be removed.

In the context of estimating gene co-expression we found that it is advisable to mean center each gene across samples. This is because the estimation of such an intercept through RUV-random can lead to the introduction of spurious correlations in some special cases. Note that the calculation of most correlation measures, like the PCC and Spearman’s correlation coefficient, involves the removal of such offsets in any case.

The RUV-random procedure for cleaning gene expression data when the interest lies in the calculation of gene-gene correlations can be summarized in four simple steps:

1. Center the data; $Y^{*}=(I_{m}-\frac {1}{m}J_{m})Y$, where $J_{m} \in \mathbb {R}^{m \times m}$ and *J*_*i*,*j*_=1 for all (*i*,*j*) and *I*_*m*_ is the *m*×*m* identity matrix.
2. Estimate $\hat {W}$ by factor analysis on $Y^{*}_{c}$, the centered expression values for the negative control genes. For example, this can be achieved using singular value decomposition (SVD) Note that this step requires the choice of an estimate for *k*, the dimensionality of the systematic noise.
3. Estimate $\hat {\alpha }$ by ridge regressing of *Y* ^∗^ on $\hat {W}$; $\hat {\alpha }=(\hat {W}' \hat {W}+\nu)^{-1} \hat {W}' Y^{*}$, where *ν*≥0 is the ridge parameter. The ridge parameter *ν* also needs to be chosen by the researcher.
4. Finally, $\hat {W}\hat {\alpha }$ can be removed from *Y* ^∗^ to obtain the noise removed, centered gene expression data.

#### Some remarks on the practical application of RUV-random in the context of gene-gene correlations

While the RUV-random procedure is easy to apply and computationally efficient, the process requires its user to make some choices concerning negative control genes and parameters. These require judgement of the analyst and may not be straightforward. The most effective way of making these choices is with the research question of interest in mind. Here, we will briefly discuss how to select negative control genes and ways to judge whether the parameter choices are suitable. A more thorough discussion on this issue, as well as the effects of wrongly specifying *ν* and $\hat {k}$, can be found in the work by Gagnon-Bartsch et al. [32].

Negative control genes are genes that are genuinely unassociated with the factor of interest and thus allow researchers to learn about the true nature of systematic noise in the data set. Ideally negative control genes should be chosen using prior biological information. For example, housekeeping genes are often a good choice [10, 18, 32]. Housekeeping genes are genes that are required for the maintenance of basic cellular activities, such as metabolism. The expression levels of these housekeeping genes are expected to be fairly constant. Published lists of housekeeping genes are available [33]. One method commonly used to discover housekeeping genes is to identify genes whose observed expression levels are fairly constant over a wide range of biological states. This is the approach of Eisenberg and Levanon. In this paper we attempt to discover negative controls using a similar strategy. Details and a cautionary discussion are provided in Additional file 1.

Judging whether parameter choices for *k* and *ν* are suitable should also be performed with the research question in mind. In particular, genes that are associated with one’s research interest and are known to be correlated with each other can be used as positive control genes. A choice of parameters that results in strong correlations between such positive control genes and weaker correlations between a random set of genes is judged successful. Researchers can determine this by looking at histogram plots as well as plots of ECDF curves of these correlations. Note that ECDF curves are often easier to interpret when comparing correlation distributions of differently sized gene sets. Additionally, Gagnon-Bartsch and Speed [10] suggest the use of relative log expression (RLE) plots for making reasonable parameter decisions. In the case of large dataset applications of RUV-random, such as the one encountered here, it is useful to modify the original RLE plots (see Additional file 1). Finally, we found principal component analysis (PCA) plots colored by known sources of unwanted variation very helpful in ascertaining whether all known systematic noise had been sufficiently removed.

### Five large microarray datasets on gene expression in the human brain

The study of gene co-expression in postmortem human brains critically informs our understanding of the molecular mechanisms of neurological disorders. However, the measurement of gene expression in postmortem brain tissue is complicated by numerous factors [34]. While the manner of death, agonal state, antemortem medication and etc. influence gene expression, differences in tissue handling and storage can also introduce complications. Because of this, careful study design and robust methods for gene expression data generation, including technical controls, are very important, however difficult to achieve. Hence, data cleaning is a crucial and necessary part of any analysis of gene expression data gained from any tissue, including human brain tissue where these effects may play an even larger role.

In this paper we use five large datasets on the transcriptome of the human brain to demonstrate the merits of the RUV-random procedure.Consent from next-of-kin was obtained in all cases for all studies and approval of all studies was granted to the authors of the primary papers that produced the data that was used in this paper. The underlying study designs of these datasets vary considerably (Table 6). One of the main differences between the studies is the number of samples analyzed for each brain. While the studies of developing and adult brains by Hawrylycz et al. and Miller et al. concentrated on obtaining a fine-scale atlas of the gene expression in few human brains, Colantuoni et al. analyzed only the prefrontal cortex in hundreds of brains. In contrast, the study by Kang et al. focused on obtaining samples from all major brains regions of a moderate number of individuals. The study of Hernandez et al. includes the largest number of brains, but examined only the prefrontal cortex and cerebellum. Another difference between the studies is their developmental focus. The studies by Kang et al. and Colantuoni et al. include brains from foetuses to adults over the age of 80 years. Examined foetuses in the Kang et al. study were as young as 4 post conception weeks (PCW) while they were at least 14 PCW in the Colantuoni et al. study. Hernandez et al. studied the gene expression in the brains of adults as well as children (1 year to 89 years). Hawrylycz et al. studied only adults (24 years to 57 years), while Miller et al. only studied foetuses (15 PCW-21 PCW). These differences in design are likely to strongly affect the level of systematic noise observed in the datasets.

**Table 6:** Study design underlying the four large microarray gene expression datasets

Study	Age Range	Platform	# Brains	# Samples	Approx.
	[youngest, oldest]				# Genes
Hawrylycz et al.	[24 years, 57 years]	Agilent 64 K	10	4063	20 000
		(custom array)			
Miller et al.	[15 PCW, 21 PCW]	Agilent	4	1310	20 000
		Human 8 ×60 K			
Kang et al.	[4 PCW, 82 years]	Affymetrix Human	57	1340	17 500
		Exon 1.0 ST			
Colantuoni et al.	[14 PCW, 80 years]	Illumina	269	269	20 000
		(custom array)			
Hernandez et al.	[1 year, 98 years]	Illumina	397	913	18 000
		HumanHT-12 V3.0			

PCW stands for post conception weeks

Most importantly, gene expression was measured using a different technology in each study, although all are microarray-based. Since the type of platform not only determines the amount of systematic noise but also influences the performance of cleaning procedures, the five analyzed datasets offer a broad testing ground to compare RUV-random to standard cleaning procedures. Note that due to the differences in technology the pre-processing protocols were adapted to the individual studies (for more information see Additional file 1).

## Additional files

Additional file 1
**Supporting information.** Further information concerning centering, application of RUV, gene prioiritization, simulations and results. (PDF 244 kb)

Additional file 2
**ECDF curves of the absolute values of correlations for the Hawrylycz et al. study.** The panels show the ECDF curves as estimated from the absolute values of the correlations of random genes (black) and EE genes (red) using the Hawrylycz et al. dataset treated with different cleaning procedures. (TIF 205 kb)

Additional file 3
**ECDF curves of the absolute values of correlations for the Miller et al. study.** The panels show the ECDF curves as estimated from the absolute values of the correlations of random genes (black) and EE genes (red) using the Miller et al. dataset treated with different cleaning procedures. (TIF 205 kb)

Additional file 4
**ECDF curves of the absolute values of correlations for the Kang et al. study.** The panels show the ECDF curves as estimated from the absolute values of the correlations of random genes (black) and EE genes (red) using the Kang et al. dataset treated with different cleaning procedures. (TIF 204 kb)

Additional file 5
**ECDF curves of the absolute values of correlations for the Colantuoni et al. dataset.** The panels show the ECDF curves as estimated from the absolute values of the correlations of random genes (black) and EE genes (red) using the Colantuoni et al. dataset treated with different cleaning procedures. (TIF 205 kb)

Additional file 6
**ECDF curves of the absolute values of correlations for the Hernandez et al. study.** The panels show the ECDF curves as estimated from the absolute values of the correlations of random genes (black) and EE genes (red) using the dataset of developing brains of the Hernandez et al. study treated with different cleaning procedures. (TIF 205 kb)

Additional file 7
**Table of arrays removed during preprocessing in each study.** (CSV 2.80 kb)
