# Supplementary material for: Systematic noise degrades gene co-expression signals but can be corrected
Source: BMC Bioinformatics. 2015 Sep 24;16:309. doi: 10.1186/s12859-015-0745-3 (PMC4583191; doi:10.1186/s12859-015-0745-3)
Supplement: Additional file 1 — Supporting information. Further information concerning centering, application of RUV, gene prioiritization, simulations and results. (PDF 244 kb) [file 12859_2015_745_MOESM1_ESM.pdf]

Supporting Information to "Systematic Noise Degrades Gene Co-Expression Signals But Can Be Corrected"

Saskia Freytag,[1,2,*] Johann Gagnon-Bartsch,[3] Terence P. Speed,[1,2,3] and Melanie Bahlo[1,2,4]

[1] *Bioinformatics Division, Walter and Eliza Hall Institute, Melbourne, Victoria, Australia*

[2] *Department of Mathematics and Statistics, University of Melbourne, Melbourne, Victoria, Australia*

[3] *Department of Statistics, University of California, Berkeley, California, USA*

[4] *Department of Medical Biology, University of Melbourne, Melbourne, Victoria, Australia*

1. APPLICATION OF RUV-RANDOM

The main principles of RUV, described in the work by Gagnon-Bartsch et al [1], also apply when the aim is the estimation of gene co-expression. However, new methods are needed to determine the effectiveness of the procedure and perform the selection of parameters.

1.1. Further Remarks on the Practical Application of RUV-random

A worked example applying RUV-random to an example gene expression dataset can be found in the vignette of the `R`-package `RUVcorr`, which can be downloaded from `https://github.com/SaskiaFreytag`. In particular, this shows how to use histograms of the correlations between pairs of genes in order to determine the size of the two parameters, ν and k.

1.1.1. Empirically Choosing Negative Control Genes

RUV requires the user to specify negative control genes. Negative control genes are genes that are assumed to have no true association with the factor of interest X. In the case of a differential expression analysis, the factor of interest is known and observed [1]. In the case of a correlation analysis, however, the notion of a "factor of interest" is less well defined. Essentially, we may think of the $X\beta$ term as representing all biological variation that we wish to preserve. In the example of this paper, we wish to preserve all biological variation that is in some way associated with epileptic

*Electronic address: `freytag.s@wehi.edu.au`

encephalopathy. Thus, a good set of negative controls would be a set of genes that can be assumed to play no role in the disease process.

One possible set of negative control genes would be so-called housekeeping genes. Housekeeping genes are involved in the basic maintenance of the cell, and their expression levels tend to be constant across a wide range of conditions. As noted in the main text, published lists of housekeeping genes are available, and one of the methods used to discover these genes is to identify genes whose expression levels are observed to be fairly constant [2].

In selecting a set of negative controls for the analyses in this paper, we elected to not use the housekeeping genes and simply used a set of genes that we observed to be fairly constant within our own datasets. Selecting our own set of negative controls in this manner also allowed us to ensure that we selected a set of genes with a representative range of expression levels. More specifically, we selected our negative controls as follows: First, we calculated the inter-quantile range of every gene. We then binned the genes according to their mean expression levels. Finally, we selected a fraction of the genes from each bin with the lowest IQR and designated these genes as our negative controls.

In the analyses of this paper, this method for selecting negative control genes seems to have produced good results. However, this method for selecting negative control genes may not be best. In particular, we have not investigated the performance of alternative sets of negative controls (e.g. published lists of housekeeping genes); such an investigation is beyond the scope of this paper. Housekeeping genes may also not be ideal as they have unknown correlation structure which may not be representative of background co-expression.

We would also like to point out some potential pitfalls associated with using this method for selecting negative controls. The primary concern is that the genes we select may not actually be negative controls. Recall our model, $Y = X\beta + W\alpha + \epsilon$. We note that there are three sources of variation that contribute to the overall observed variation in gene expression levels the variation of interest ($X\beta$), the unwanted variation ($W\alpha$), and random noise (ϵ). By selecting genes that show an overall low level of variation, we hope to exclude genes that exhibit any variation of interest. However, it is also possible that we might select genes that simply have an unusually low level of unwanted variation or random noise. A more pernicious problem may arise if X and W are negatively correlated. In such cases, the variation of interest and the unwanted variation may partially cancel out and thus may prevent the effective removal of the systematic noise. Unfortunately, it is impossible to assess whether these caveats apply in our real datasets.

Finally, note that RUV-random assumes a mixed-effects model, in which the $W\alpha$ term is mod-

eled as random. The negative controls are used to estimate the covariance of this term. However, by systematically selecting genes observed to have a low level of variation as our negative controls, we may bias our estimate of the covariance of $W\alpha$.

1.1.2. RLE Plots for Large Data Sets

RLE plots are valuable graphical tools for estimating parameters required for the application of RUV-random. Unfortunately, for large data sets RLE plots loose their appeal, as it is impractical to compare hundreds or even thousands of boxplots. Here, we present a new RLE plot that neatly summarizes the information for all samples into one plot and additionally offers the possibility of adding information on known sources of systematic noise.

The original RLE plot is a boxplot of the devation from the median gene expression calculated for each gene in a sample. Using the width of the boxplot against the median deviation, it is possible to plot all samples in the same plot. While this still allows the easy identification of outliers, it has the added benefit of providing a study-wide perspective. In particular, samples can be colored according to the levels of a known source of systematic noise (compare Figure 1). This is implemented in the function `RLEPlot` in the package `RUVcorr`.

FIG. 1: **Novel RLE plots for all samples in the Colantuoni et al dataset.**
The left-hand panel shows novel RLE plot for RUV-random treated data and the right-hand panel displays the untreated data. The samples are colored by which batch they belong to. The corresponding legend can be found on the far left. For a more thorough description of the novel RLE plot see the text.

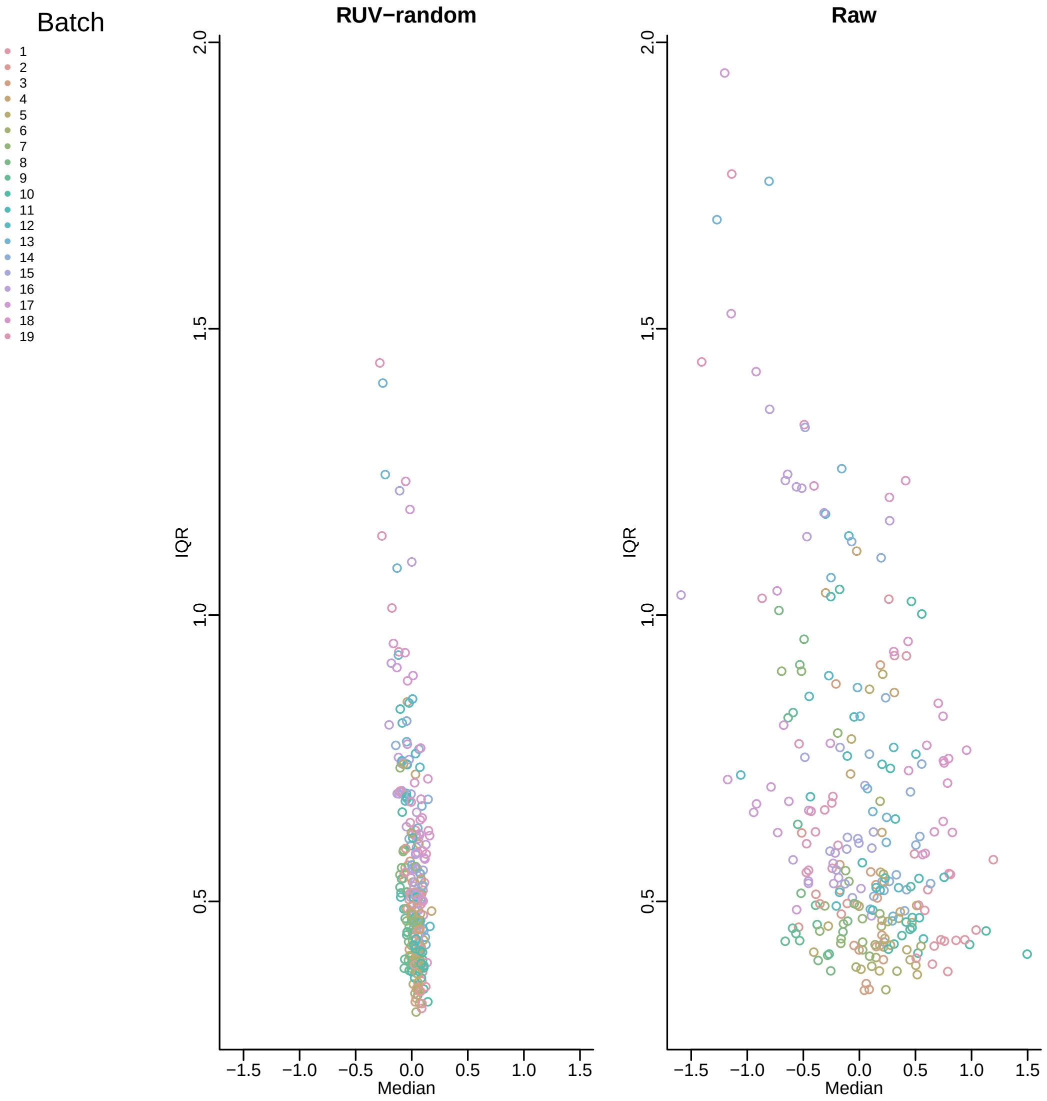

2. FIVE LARGE DATASETS ON GENE EXPRESSION IN THE HUMAN BRAIN

We downloaded the raw (i.e. non-normalized) data for each study. Data for the Colantuoni et al study [3] (acession number: GSE30272) and the Hernandez et al study [4] (acession number: GSE36192) can be accessed via NCBI's Gene Expression Omnibus. The data for the studies by Hawrylycz et al [5] can be found on the webpage of the Allen Institute for Brain Science [6] . The data for the studies by Miller et al and Kang et al can be accessed through BrainSpan [7].

2.1. Data Preprocessing

Due to the differences in study design and technology preprocessing protocols varied. However, for all datasets we applied background correction [8] and then quantile-normalization [9] or in case of the Colantuoni et al dataset loess-normalization [10]. In the following we briefly describe the preprocessing steps taken for each study.

2.1.1. Datasets by Hawrylycz et al (Allen Human Brain Atlas) and Miller et al (Developing Human Brain Atlas)

Both studies relied on Agilent technology to measure gene expression (see [11] for more details). Analysis was conducted using the `R` package `limma`. Following the protocol established by Hawrylycz et al we identified arrays that failed at least one of the Agilent criteria specified in their technical paper [12]. These arrays were further investigated using spot images, MA-plots, PCA-plots as well as array density plots. Thus, we identified 185 abnormal or failed arrays for the Hawrylycz et al and 82 arrays in the Miller et al dataset (see S1 Table for the names of the removed arrays). These were consequently removed from further analysis. We then proceeded to apply the standard normalization approaches. Since the datasets in this study included some replicate samples. We averaged the samples for these. We then removed transcripts that were non-human or not annotated as well as all technical controls. Transcripts with multiple measurements were represented by the measurement with the largest median value across all samples. Finally, we $\log_2$ transformed all gene expression values.

2.1.2. Dataset by Kang et al

In this study, gene expression was measured using the Affymertix Human Exon 1.0 ST array. Analysis was conducted with the `R` package `aroma.affymetrix` [13]. First we checked array density plots and spot images identifying 11 abnormal or failed arrays that we removed from further analysis (see S1 Table for the names of the removed arrays). We then normalized the data using the approaches outlined above. After this probe level modelling was applied to the differently treated datasets including the untreated data. We then removed transcripts that were non-human or not annotated as well as all technical controls introduced by the manufacturer. Transcripts with multiple measurements were represented by the measurement with the largest median value across all samples. Finally, we $\log_2$ transformed all gene expression values.

2.1.3. Dataset by Colantuoni et al

In this study, gene expression was measured using a custom Illumina two-color microarray with a common reference. Using the R package `limma` [14] we calculated the M values for further analysis, which is standard procedure when dealing with two-color microarrays with a common reference. These were used to create array density plots as well as PCA plots. In combination with MA-plots for each sample, these plots helped us to identify three failed or abnormal arrays which were consequently removed from further analysis (see S1 Table for the names of the removed arrays). We then proceeded to apply standard normalization procedures for comparison with RUV-random. We removed transcripts that were non-human or not annotated as well as all tiling and technical controls. Furthermore, transcripts with multiple measurements were represented by the measurement with the largest median value across all samples, a standard method. Finally, we $\log_2$ transformed all gene expression values.

2.1.4. Dataset by Hernandez et al

In this study, gene expression was measured using an Illumina HumanHT-12 V3.0 beadchip. Analysis was conducted with the R package `limma`. First we checked array density plots, MA-plots and PCA-plots identifying 5 abnormal or failed arrays that we removed from further analysis (see S1 Table for the names of the removed arrays). We then normalized the data using the approaches outlined above. We then removed transcripts that were non-human or annotated as 'bad matches' by the manufacturer. Transcripts with multiple measurements were represented by the measurement with the largest median value across all samples. Finally, we $\log_2$ transformed all gene expression values.

3. PRIORITZING EE CANDIDATE GENES

In 2014, Oliver et al provided new insights into the mechanism of EE using gene prioritization [15]. In this paper, we follow their approach with few modifications and some updates concerning known EE genes and candidates.

3.1. Gene Prioritization Method

Candidate genes are prioritized when they are co-expressed with at least one known EE gene. Thus, for each candidate and each known EE genes combination, co-expression needs to be established. Oliver et al used the PCC (or a weighted version introduced by Horvath [16] in case of multiple samples per genes) in order to establish the amount of co-expression. In order to avoid declaring spuriously correlated genes co-expressed, they used a thresholding approach. We also make use of the PCC combined with a thresholding approach. Here, we set the threshold for an absolute value of the PCC that corresponds to the top 0.2 of ranked random genes. These random genes were ranked according to their maximum absolute correlation with any of the known EE genes. This process was repeated 1000 times and the average maximum absolute correlation was calculated and set as a threshold. Note that the size of the randomly selected genes corresponded to the number of candidates in the various datasets.

3.2. Known EE Genes and Candidates

The list of known EE genes used in the Oliver et al paper was updated from 29 known EE genes to 33 confirmed EE genes (as known in September 2014). Candidate genes included all candidates used in the Oliver et al paper, well-established genes for other types of epilepsies and recent significant findings from the ILAE meta-analysis of genome-wide association studies [17]. Genes 500kb up- or downstream of the location of these findings were included in the list. Table I gives the number of candidates and known EE genes found in each dataset. Note that for the application of RUV-random, all known EE genes and candidates were excluded from being selected as negative control genes.

TABLE I: **Number of known EE genes (total 33) and candidates (total 223) in the five datasets.**

**Study**	**# Known EE Genes**	**# Candidate EE Genes**
Hawrylycz et al	30	201
Miller et al	30	203
Kang et al	30	206
Hernandez et al	33	191
Colantuoni et al	27	174

4. SIMULATION

The simulation framework is based on the RUV linear model, which is described in the Section Methods and Materials of the accompanying paper. For the purposes of investigating the performance of different cleaning procedures in the context of gene-gene correlations, it is necessary to known the true underlying correlation structure between the simulated genes. Therefore, the fundamental concept of this simulation is that $\Sigma = \mathrm{Cor}(X\beta)$ represents the genuine gene-gene correlation structure induced by the biological signal. This setup allows control of the average absolute value of the gene-gene correlations through the dimensionality of X and β, p. In the following we explain the simulation setup in detail.

4.1. Simulation Setup

For the simulation the parameters of the RUV linear model where simulated in the following manner:

- X is a $m \times p$ matrix with $x_{i,j} \sim N(0,1)$ for $i = 1, ..., m$ and $j = 1, ..., p$.
- β is a $p \times n$ matrix with

$$\beta_{i,j} \begin{cases} \sim \frac{2}{\sqrt{p}} Uni(-1,1) & \text{for } j = 1, ...p \text{ and } j = 1, ..., (p - n_c) \\ = 0 & \text{for } j = 1, ...p \text{ and } j = (p - n_c + 1), ..., p \end{cases}$$

  where n_c is the number of negative control genes.
- W is a $m \times k$ matrix with $w_{i,j} \sim N(0,1)$ for $i = 1, ...m$ and $j = 1, ..., k$.
- α is a $k \times n$ matrix with $\alpha_{i,j} \sim (2\tilde{\alpha}/\sqrt{k})Uni(-1,1)$ for $i = 1, ..., k$ and $j = 1, ..., n$, where $\tilde{\alpha}$ is the level of the systematic noise.
- ϵ is a $m \times n$ matrix with $\epsilon_{i,j} \sim N(0, \tilde{\epsilon})$ for $i = 1, ..., m$ and $j = 1, ..., n$, where $\tilde{\epsilon}$ is associated variance.

Note that we define $\Sigma_{i,j} = 0$ when either $i = (p - n_c + 1), ..., p$ or $j = (p - n_c + 1), ..., p$. To simulate correlation between X and W define $\mathfrak{X} = \big(X|W\big)$ is $m \times (k + p)$ matrix with rows $\mathfrak{X}_i \sim N(0, \Sigma_{\mathfrak{X}})$ for $i = 1, .., m$. The covariance matrix is defined by $\Sigma_{\mathfrak{X}} = \left(\begin{smallmatrix} I_{p\times p} & L \\ L^T & I_{k\times k} \end{smallmatrix}\right)$

where

$$L_{k\times p} = \begin{pmatrix} I_{g\times g} & 0 & \dots & 0 \\ 0 & & \ddots & \\ \vdots & & \ddots & \\ 0 & \dots & \dots & 0 \end{pmatrix}$$

with g denoting the dimensionality of the shared subspace of X and W. Hence, the parameter g controls the average correlation between the columns of X and W. If g is increased then the average correlation increases.

In our `R`-package `RUVcorr` the sizes of all parameters presented above can be varied. For the simulation scenarios presented here we used the following values, unless they were varied for specific simulations:

- Number of samples $m = 1000$
- Number of genes $n = 2500$ of which $n_c = 2000$ are negative control genes
- Level of systematic noise $\tilde{\alpha} = 2$
- Variance of random noise $\tilde{\epsilon} = 0.01$
- Dimensionality of systematic noise $k = 10$
- Dimensionality of factor of interest $p = 5$
- No correlation between X and W

Note that in the application of RUV-random generally all 2000 simulated negative control genes were used. We also assumed that the values of k and ν were known.

4.2. Further Simulation Scenarios

We investigated the performance of RUV-random when the systematic noise level was varied (compare Table II). Interstingly, the performance of RUV-random in the simulations is absolutely unchanged no matter how much systematic noise is present in the data.

TABLE II: **Performance of RUV-random when when the level of systematic noise ($\tilde{\alpha}$) is varied.** The performance was measured using the prercentage of estimates with the wrong sign (WS). While FN^2 refers to a measure similar to the squared Frobenius norm (FN^2) (explained in the text), which should be close to 0. All parameter choices and details of the simulation can be found in Section 4 4.1. All standard deviation for FN^2 were < 0.001.

$\tilde{\alpha}$	% WS	FN^2
1	0.7	0.005
2	0.7	0.005
3	0.7	0.005
4	0.7	0.005

4.3. Combining RUVcorr with Other Cleaning Procedures

When combining RUV-random with other cleaning approaches we had to find the optimal choice for the parameters k and ν first. Interestingly, the optimal choices for k were generally very large indicating that the application of QN or BC introduced new systematic noise that RUV-random struggles to remove. Generally, RUV-random on its own did much better than combining approaches (compare Table III). However, it needs to be remembered that we did not simulate measured background noise.

TABLE III: **Performance of RUV-random on basic simulation scenario when combined with other cleaning approaches.** Results are based on 1000 simulations each with 500 genes with known correlation structure. The performance is assessed by a measure similar to the squared Frobenius norm (FN^2) (explained in the main paper) and the percentage of estimated with the wrong sign (WS). The corrsponding simulation parameters are given in Section 4 4.1. The parameters, ν and k, were chosen to optimize results. All standard deviation for FN^2 were < 0.001.

**Procedure**	**% WS**	**FN^2**
RUV-random	0.7	0.005
BC+RUV-random	1.5	0.014
BC+QN+RUV-random	4.5	0.046

4.4. Incorrectly Specified Negative Control Genes

We simulated the effect of incorrectly specified negative control genes. For this different proportions of our 2000 negative controls were selected to be genes that were simulated to have

co-expression with other genes. Figure 2 demonstrates that even 50% incorrectly specified negative control genes had little effect on the performance of RUV-random. Indeed, it seems as long as there are enough genuine negative control genes RUV-random perfoms well. Thus, we investigated how many negative control genes are necessary for RUV-random to perform well. Figure 3 shows that with a number twice as large as k correlations estimated from the RUV-random corrected simulated data are already very close to the truth. However, we would still strongly recommend using as many negative controls as possible.

FIG. 2: **Performance of RUV-random when the proportion of misspecified negative control genes is increased.** The performance is assessed using the proportion of estimates with the wrong sign (WS). The performance of RUV-random is compared to using the raw data. The corrsponding simulation parameters are given in Section 4 4.1.

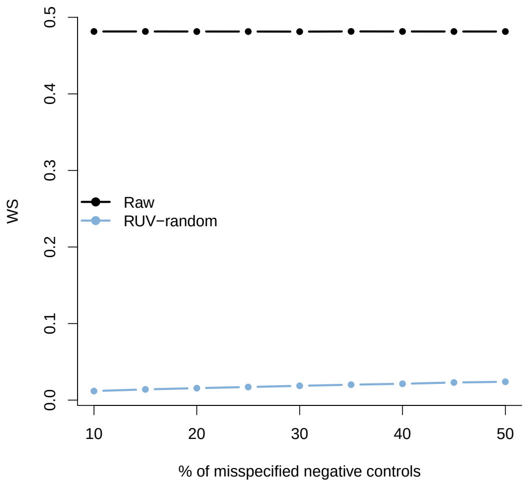

FIG. 3: **Performance of RUV-random with small numbers of negative control genes.** The performance is assessed using the proportion of correlatione estimates with the wrong sign (WS). The errorbars show the size of the standard errors. The corrsponding simulation parameters are given in Section 4 4.1.

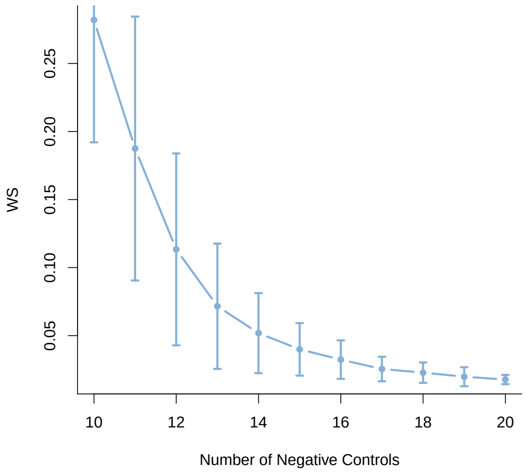

5. REAL DATA

In this Section we briefly comment on the application of RUV-random to the real datasets and we provide some more information on the results of the prioritzation of the candidate EE genes.

5.1. Application of RUV-random to the Real Datasets

As discussed earlier, the application of RUV-random requires the researcher to choose various parameters. For this paper we limit ourselves to giving our parameter choices for each dataset (compare Table IV), but do not provide detailed explanation for our choices. Briefly, we tried to select $\hat{k}$ and ν in a way to avoid over-correction and removal of the factor of interest, while at the same trying to ensure that all systematic noise from known sources was removed. To this end we used the methods discussed in the Section Methods and Materials of the paper and Section 1 1.1.

TABLE IV: **Parameter choices for RUV-random as used for the different datasets.**

**Study**	$\hat{k}$	ν
Hawrylycz et al	5	50 000
Miller et al	4	500 000
Colantuoni et al	3	25 000
Kang et al	3	10 000
Hernandez et al	3	30 000
BC version of Hernandez et al	1	20 000

For all datasets we used 4000 empirically chosen negative control genes for RUV-random. Note that the performance of RUV-random is determined by the absolute number of negative control genes and not by the proportion of negative control genes to all genes in the dataset.

5.2. Results of Prioritization of EE Candidate Genes

TABLE V: **EE candidate genes prioirtized in all RUVcorr treated datasets.** For each gene we looked for recent publications that confirmed its involvement in EE.

**Gene**	**References of Recent Confirmation**
GNAO1	Kehrl et al, 2014[18]
DNM1	EuroEPINOMICS-RES Consortium et al, 2014 [19]
GABRA1	Carvill et al, 2014 [20]
YWHAG	
MAST1	
NBEA	
KCNB1	Torkamani et al, 2014 [21]

[1] Gagnon-Bartsch, J.A., Speed, T.P.: Using control genes to correct for unwanted variation in microarray data. Biostatistics **13**(3), 539–552 (2012)

[2] Eisenberg, E., Levanon, E.Y.: Human housekeeping genes, revisited. Trends in Genetics **29**(10), 569–574 (2013)

[3] Temporal Dynamics and Genetic Control of Transcription in the Human Prefrontal Cortex. `http://www.ncbi.nlm.nih.gov/geo/query/acc.cgi?acc=GSE30272`. Accessed: 2014-09-23

[4] North American Brain Expression Consortium and UK Human Brain Expression Database: Gene Expression. http://www.ncbi.nlm.nih.gov/geo/query/acc.cgi?acc=GSE36192. Accessed: 2014-10-15

[5] Hawrylycz, M.J., Lein, E.S., Guillozet-Bongaarts, A.L., Shen, E.H., Ng, L., Miller, J.A., van de Lagemaat, L.N., Smith, K.A., Ebbert, A., Riley, Z.L., *et al.*: An anatomically comprehensive atlas of the adult human brain transcriptome. Nature **489**(7416), 391–399 (2012)

[6] Allen Institute for Brain Science. http://human.brain-map.org/static/download. Accessed: 2014-06-30

[7] BrainSpan. http://www.ncbi.nlm.nih.gov/geo/query/acc.cgi?acc=GSE36192. Accessed: 2014-07-1

[8] Irizarry, R.A., Hobbs, B., Collin, F., Beazer-Barclay, Y.D., Antonellis, K.J., Scherf, U., Speed, T.P.: Exploration, normalization, and summaries of high density oligonucleotide array probe level data. Biostatistics **4**(2), 249–264 (2003)

[9] Bolstad, B.M., Irizarry, R.A., Åstrand, M., Speed, T.P.: A comparison of normalization methods for high density oligonucleotide array data based on variance and bias. Bioinformatics **19**(2), 185–193 (2003)

[10] Ritchie, M.E., Silver, J., Oshlack, A., Holmes, M., Diyagama, D., Holloway, A., Smyth, G.K.: A comparison of background correction methods for two-colour microarrays. Bioinformatics **23**(20), 2700–2707 (2007)

[11] Allen Institute for Brain Science: Technical white paper: Microarray survey. Technical report (October 2013)

[12] Allen Institute for Brain Science: Technical white paper: Microarray data normalization. Technical report (March 2013)

[13] Bengtsson, H., Simpson, K., Bullard, J., Hansen, K.: aroma.affymetrix: A generic framework in R for analyzing small to very large Affymetrix data sets in bounded memory. Technical Report 745, Department of Statistics, University of California, Berkeley (February 2008)

[14] Ritchie, M.E., Phipson, B., Wu, D., Hu, Y., Law, C.W., Shi, W., Smyth, G.K.: limma powers differential expression analyses for rna-sequencing and microarray studies (2015)

[15] Oliver, K.L., Lukic, V., Thorne, N.P., Berkovic, S.F., Scheffer, I.E., Bahlo, M.: Harnessing gene expression networks to prioritize candidate epileptic encephalopathy genes. PloS one **9**(7), 102079 (2014)

[16] Horvath, S.: Weighted Network Analysis: Applications in Genomics and Systems Biology, 1st edn. Springer, ??? (2011)

[17] Speed, D., Hoggart, C., Petrovski, S., Tachmazidou, I., Coffey, A., Jorgensen, A., Eleftherohorinou, H., De Iorio, M., Todaro, M., De, T., *et al.*: A genome-wide association study and biological pathway analysis of epilepsy prognosis in a prospective cohort of newly treated epilepsy. Human molecular genetics **23**(1), 247–258 (2014)

[18] Kehrl, J.M., Sahaya, K., Dalton, H.M., Charbeneau, R.A., Kohut, K.T., Gilbert, K., Pelz, M.C., Parent, J., Neubig, R.R.: Gain-of-function mutation in gnao1: A murine model of epileptiform encephalopathy (eiee17)? Mammalian Genome **25**(5-6), 202–210 (2014)

[19] Phenome, E., Consortium, E.-R., Consortium, E., *et al.*: De novo mutations in synaptic transmission genes including dnm1 cause epileptic encephalopathies. The American Journal of Human Genetics **95**(4), 360–370 (2014)

[20] Carvill, G.L., Weckhuysen, S., McMahon, J.M., Hartmann, C., Møller, R.S., Hjalgrim, H., Cook, J., Geraghty, E., ORoak, B.J., Petrou, S., *et al.*: Gabra1 and stxbp1: Novel genetic causes of dravet syndrome. Neurology **82**(14), 1245–1253 (2014)

[21] Torkamani, A., Bersell, K., Jorge, B.S., Bjork, R.L., Friedman, J.R., Bloss, C.S., Cohen, J., Gupta, S., Naidu, S., Vanoye, C.G., *et al.*: De novo kcnb1 mutations in epileptic encephalopathy. Annals of neurology **76**(4), 529–540 (2014)
