# Supplementary figures and images for: Systematic noise degrades gene co-expression signals but can be corrected
Source: BMC Bioinformatics. 2015 Sep 24;16:309. doi: 10.1186/s12859-015-0745-3 (PMC4583191; doi:10.1186/s12859-015-0745-3)

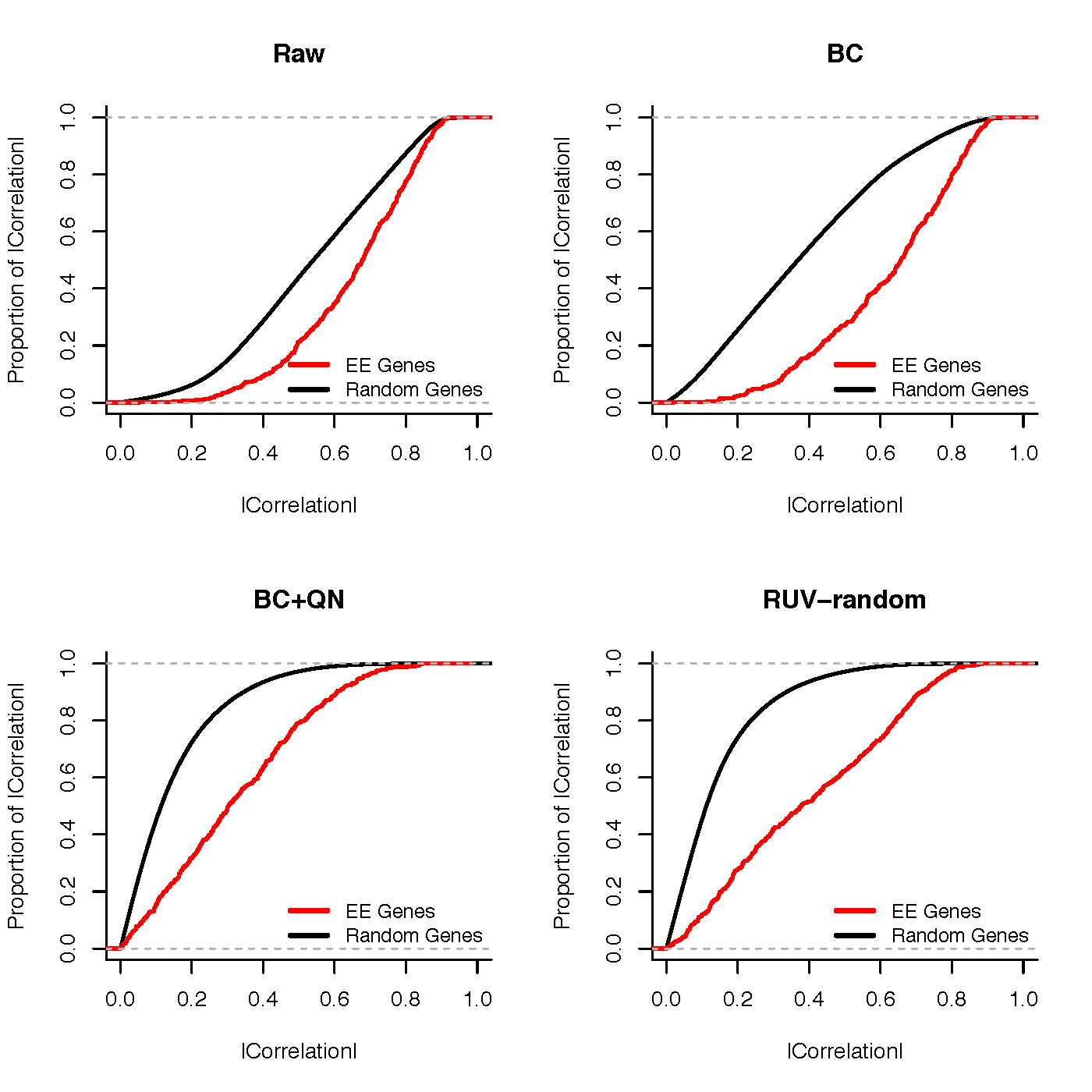

Supplement: Additional file 2 — ECDF curves of the absolute values of correlations for the Hawrylycz et al. study. The panels show the ECDF curves as estimated from the absolute values of the correlations of random genes (black) and EE genes (red) using the Hawrylycz et al. dataset treated with different cleaning procedures. (TIF 205 kb) [file 12859_2015_745_MOESM2_ESM.tif]

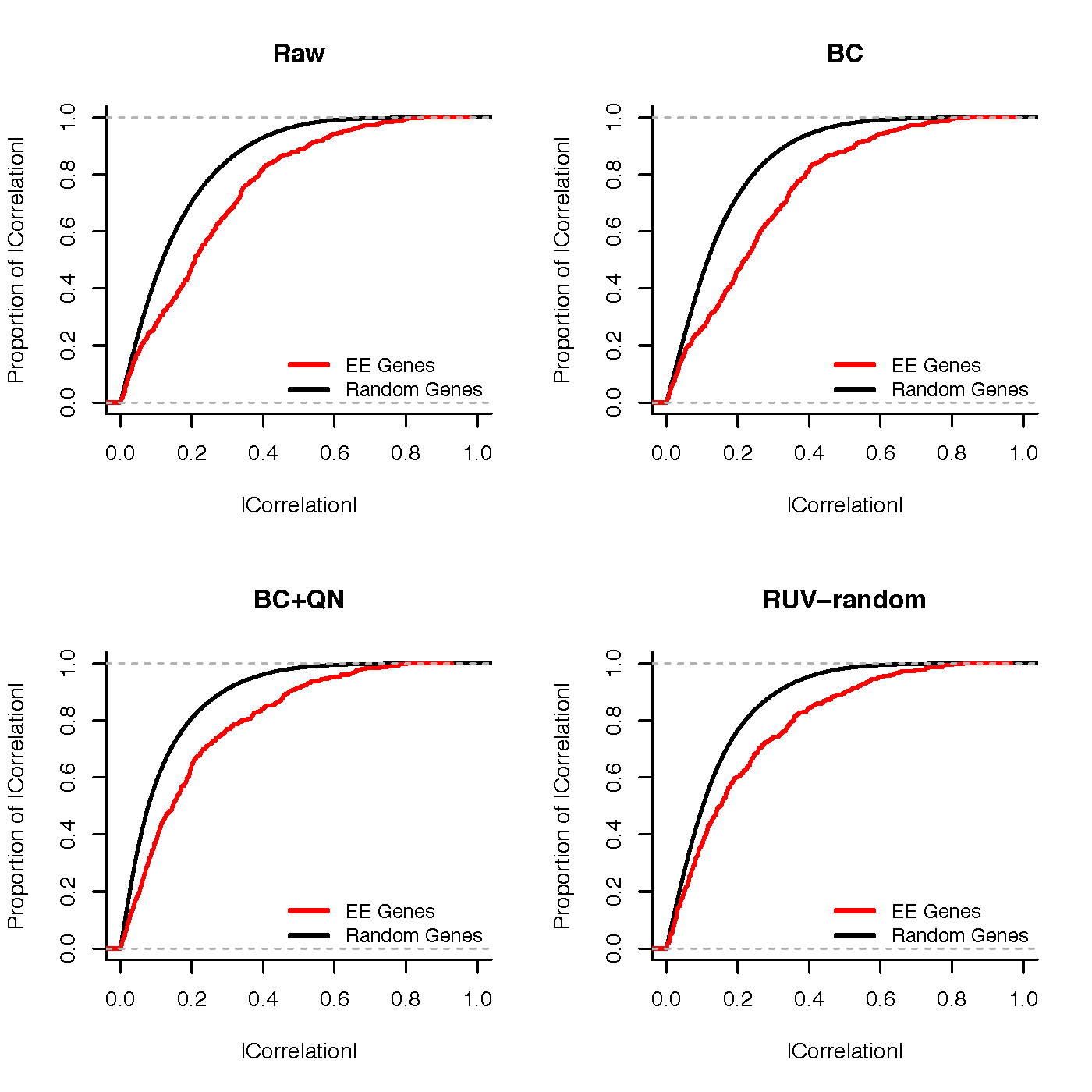

Supplement: Additional file 3 — ECDF curves of the absolute values of correlations for the Miller et al. study. The panels show the ECDF curves as estimated from the absolute values of the correlations of random genes (black) and EE genes (red) using the Miller et al. dataset treated with different cleaning procedures. (TIF 205 kb) [file 12859_2015_745_MOESM3_ESM.tif]

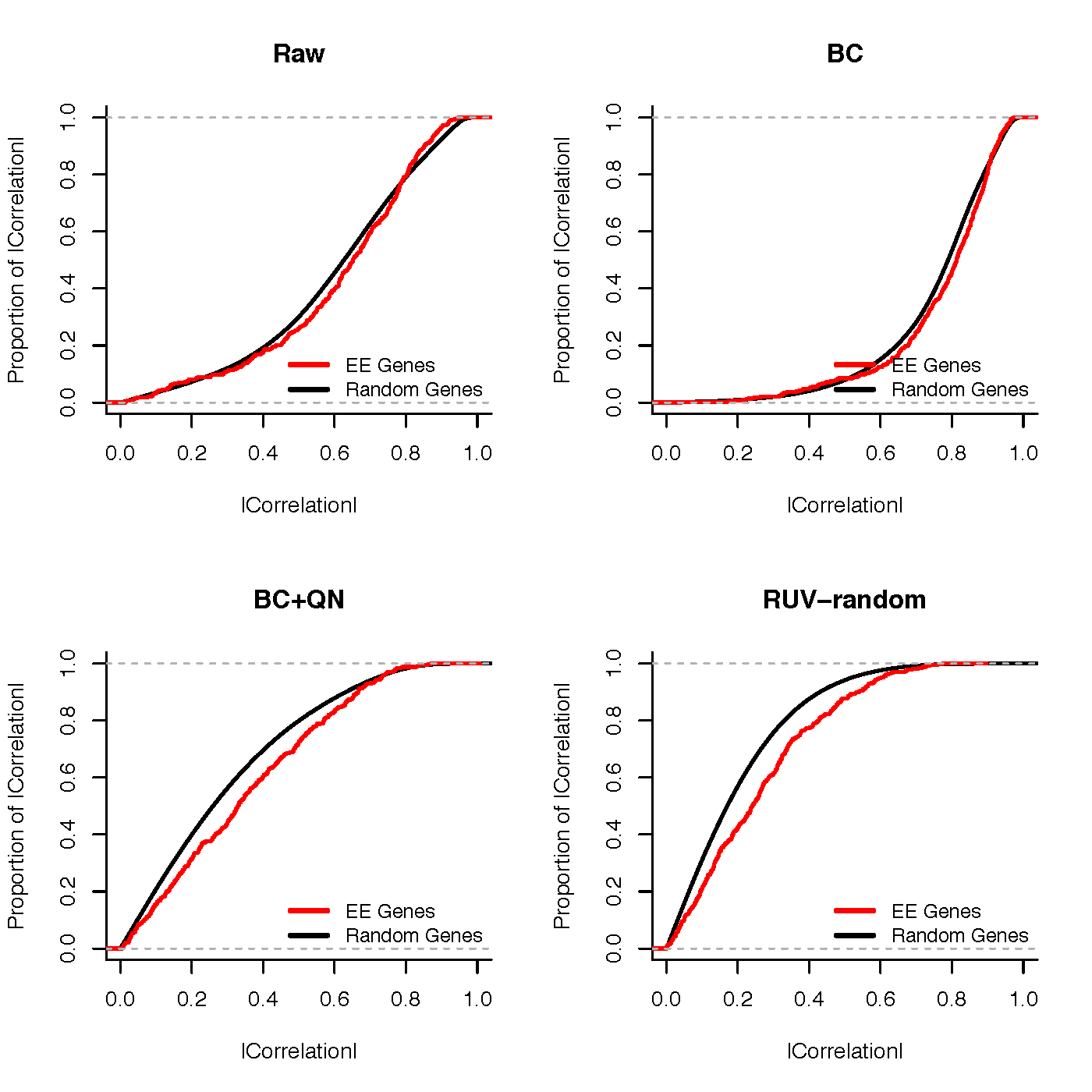

Supplement: Additional file 4 — ECDF curves of the absolute values of correlations for the Kang et al. study. The panels show the ECDF curves as estimated from the absolute values of the correlations of random genes (black) and EE genes (red) using the Kang et al. dataset treated with different cleaning procedures. (TIF 204 kb) [file 12859_2015_745_MOESM4_ESM.tif]

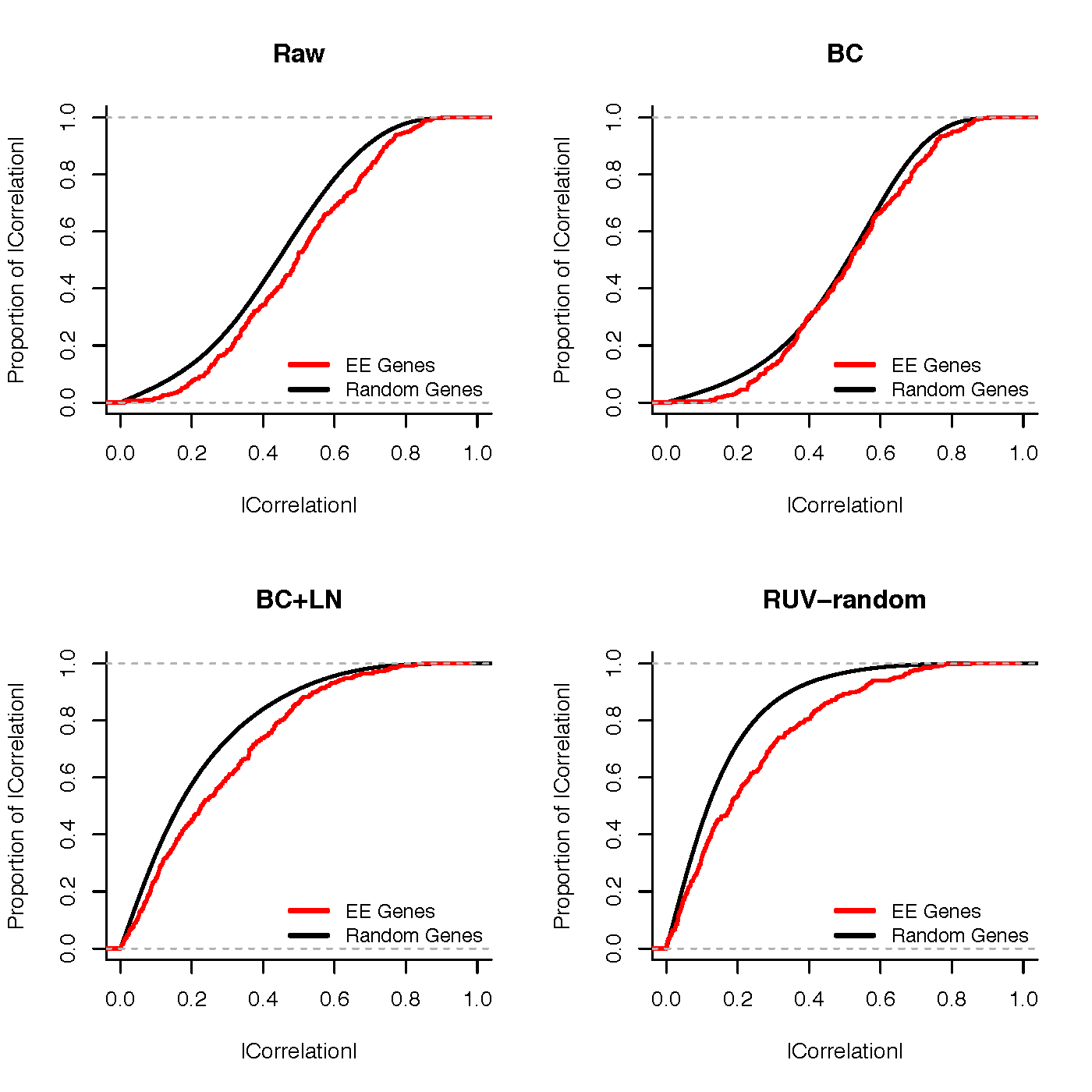

Supplement: Additional file 5 — ECDF curves of the absolute values of correlations for the Colantuoni et al. dataset. The panels show the ECDF curves as estimated from the absolute values of the correlations of random genes (black) and EE genes (red) using the Colantuoni et al. dataset treated with different cleaning procedures. (TIF 205 kb) [file 12859_2015_745_MOESM5_ESM.tif]

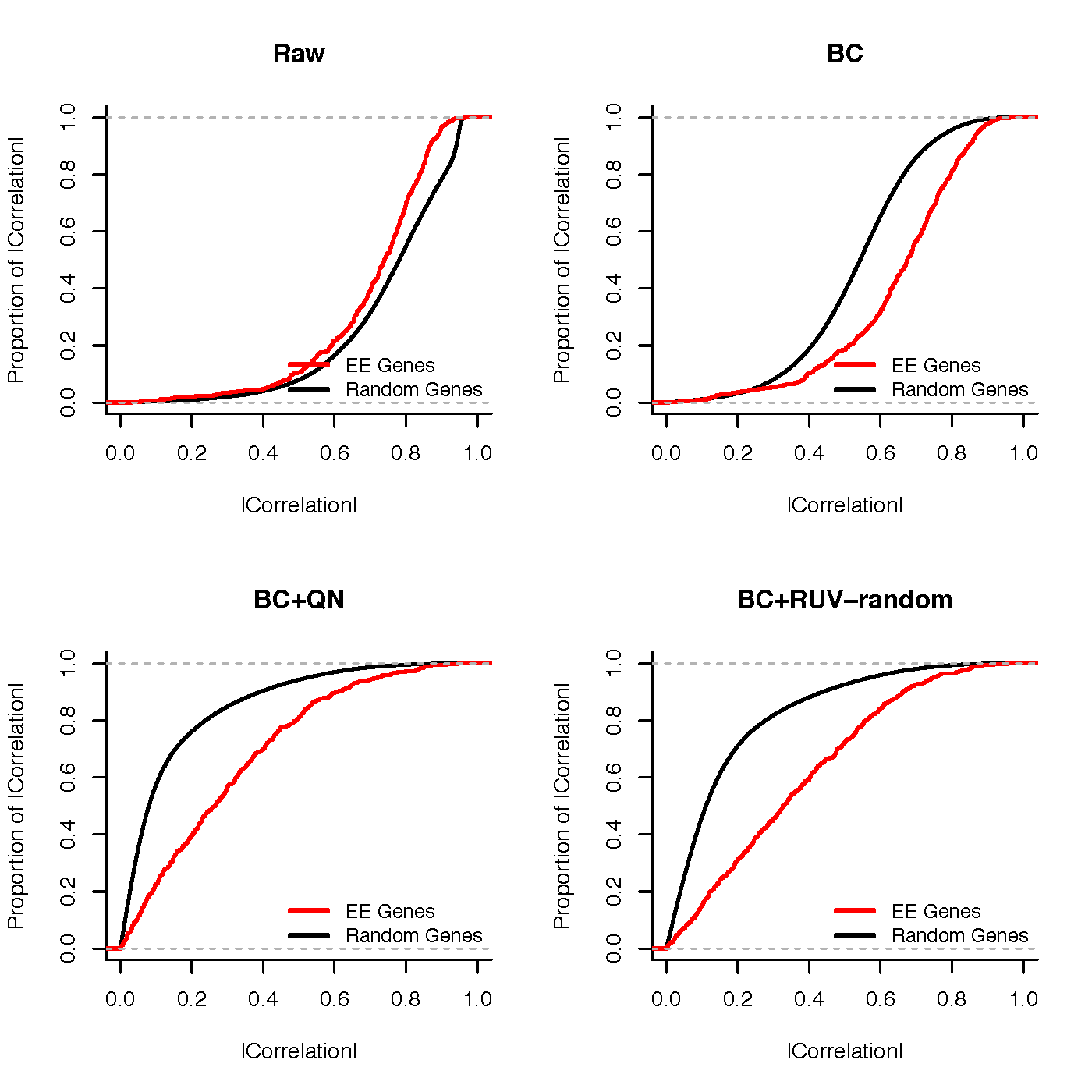

Supplement: Additional file 6 — ECDF curves of the absolute values of correlations for the Hernandez et al. study. The panels show the ECDF curves as estimated from the absolute values of the correlations of random genes (black) and EE genes (red) using the dataset of developing brains of the Hernandez et al. study treated with different cleaning procedures. (TIF 205 kb) [file 12859_2015_745_MOESM6_ESM.tif]
